# Quantitative global sensitivity analysis of a biologically based dose-response pregnancy model for the thyroid endocrine system

**DOI:** 10.3389/fphar.2015.00107

**Published:** 2015-05-27

**Authors:** Annie Lumen, Kevin McNally, Nysia George, Jeffrey W. Fisher, George D. Loizou

**Affiliations:** ^1^Division of Biochemical Toxicology, National Center for Toxicological Research, U.S. Food and Drug AdministrationJefferson, AR, USA; ^2^Health and Safety LaboratoryBuxton, UK; ^3^Division of Bioinformatics and Biostatistics, National Center for Toxicological Research, U.S. Food and Drug AdministrationJefferson, AR, USA

**Keywords:** global sensitivity analysis, BBDR, PBPK, pregnancy, thyroid, thyroid hormones, iodide, modeling

## Abstract

A deterministic biologically based dose-response model for the thyroidal system in a near-term pregnant woman and the fetus was recently developed to evaluate quantitatively thyroid hormone perturbations. The current work focuses on conducting a quantitative global sensitivity analysis on this complex model to identify and characterize the sources and contributions of uncertainties in the predicted model output. The workflow and methodologies suitable for computationally expensive models, such as the Morris screening method and Gaussian Emulation processes, were used for the implementation of the global sensitivity analysis. Sensitivity indices, such as main, total and interaction effects, were computed for a screened set of the total thyroidal system descriptive model input parameters. Furthermore, a narrower sub-set of the most influential parameters affecting the model output of maternal thyroid hormone levels were identified in addition to the characterization of their overall and pair-wise parameter interaction quotients. The characteristic trends of influence in model output for each of these individual model input parameters over their plausible ranges were elucidated using Gaussian Emulation processes. Through global sensitivity analysis we have gained a better understanding of the model behavior and performance beyond the domains of observation by the simultaneous variation in model inputs over their range of plausible uncertainties. The sensitivity analysis helped identify parameters that determine the driving mechanisms of the maternal and fetal iodide kinetics, thyroid function and their interactions, and contributed to an improved understanding of the system modeled. We have thus demonstrated the use and application of global sensitivity analysis for a biologically based dose-response model for sensitive life-stages such as pregnancy that provides richer information on the model and the thyroidal system modeled compared to local sensitivity analysis.

## Introduction

Computational modeling approaches, such as physiologically based pharmacokinetic/pharmacodynamic (PBPK/PD) and biologically based dose-response (BBDR) models, are currently being well embraced for the study of the system-compound interactions and are increasingly used in regulatory decision making for both pharmaceuticals and environmental chemicals (Zhao et al., 2011; Huang et al., 2013; McLanahan et al., 2014). Sufficiently robust BBDR and PBPK/PD models that are developed to include the key physiological traits of the organism under study are able to describe the pharmacokinetic disposition of compounds quantitatively and also provide relevant mechanistic insights of the system-compound interaction based on the biological mode-of-action. The use of PBPK models includes the prediction of internal dose metrics and target organ specific exposure levels that correspond to a known external dose and/or exposure (Clewell et al., 2003, 2007; Merrill et al., 2005). Pharmacodynamic models allow the characterization of relevant mechanism-based internal dose response relationships (Andersen et al., 1997; Felmlee et al., 2012; Gentry et al., 2014). BBDR models can encompass multiple compound-specific PBPK submodels in addition to the pharmacodynamic submodel components, linking external exposure to a quantifiable biological response for an array of doses (Conolly and Butterworth, 1995; Setzer et al., 2001; McLanahan et al., 2008; Fisher et al., 2013; Lumen et al., 2013). The application of these models range from supporting risk assessment and public health decisions to identifying data gaps and research needs to further basic science (Doerge et al., 2008; Kenyon et al., 2008; Tan et al., 2012). Such models also offer a useful framework for integrating available data from diverse platforms, including *in vitro* and *in vivo* studies, and offer means to scale and extrapolate across species to humans and to sensitive life-stages, such as pregnancy.

Recently, we developed a BBDR model for the hypothalamus-pituitary-thyroid (HPT) axis in an average near-term pregnant woman and the fetus (Lumen et al., 2013). The model described the disposition kinetics of dietary iodide during pregnancy followed by the pharmacodynamic description of the organification of inorganic iodide in the maternal and fetal thyroid for the synthesis and secretion of thyroid hormones. The BBDR-HPT axis model also described the physiologic disposition of the thyroid hormones accounting for the placental transfer of maternal thyroxine to the fetus in addition to inorganic iodide transfer for the sustenance of the developing fetal thyroid's function and its neurodevelopmental needs. Disturbances in the HPT axis during pregnancy have been shown to be associated with neurodevelopmental effects in the fetus in utero and the neonate after birth (Man et al., 1991; Haddow et al., 1999; Kooistra et al., 2006; Taylor et al., 2014). Iodide deficiency is a major cause for such disturbances, and exposure to thyroid-active environmental chemicals, such as perchlorate, thiocyanate, and nitrate, that competitively inhibit the thyroidal uptake of iodide may predispose sensitive individuals to further alterations in thyroid endocrine homeostasis. The mode-of-action based model was used to predict quantitatively alterations in maternal and fetal serum thyroid hormone levels at steady state for combinatorial scenarios of iodide nutritional status and environmental exposure levels for perchlorate, demonstrating its utility as a risk assessment tool. The confidence in the model's ability to evaluate thyroid axis disruption due to perchlorate exposure lies strongly in the robustness of the model's description of the thyroid endocrine function and is the focus of our current work.

Although these models have certain strengths, they are usually complex in nature with a large-set of input parameters that are calibrated to available data sets for certain input conditions and also involve simplifying assumptions of the biological system that it emulates. Together, these contribute to uncertainties in the model and model predictions. The model developed in Lumen et al. (2013) is deterministic in nature. The current work focuses on methodologies and their use for evaluating the sources and contributions to uncertainties in the BBDR-HPT axis pregnancy model. Typically, a sensitivity analysis is employed to test the model robustness with respect to parameter uncertainties and investigate the influence of input parameters on model performance. Several different approaches can be followed for performing model sensitivity analysis (Sobol, 1993, 2001; Campolongo and Saltelli, 1997; Saltelli et al., 1999, 2004; Oakley and O'Hagan, 2004; Loizou et al., 2008). The most commonly used approach in such physiologically based modeling is the local or one-at-a-time sensitivity analysis. Local sensitivity analysis is performed by perturbing parameters one-at-a-time, typically by increasing or decreasing values by a small percentage and monitoring the effects on the model output relative to the change in the input parameter (Plowchalk et al., 1997; Rietjens et al., 2011). Local sensitivity analysis offers a more straight-forward and computationally inexpensive means to perform sensitivity analysis on these elaborate models. However, it does not consider the effects of simultaneous variations in multiple input parameters on model output and neglects any parameter interactions (Sobol, 1993, 2001; Campolongo and Saltelli, 1997; Saltelli et al., 1999, 2004; Oakley and O'Hagan, 2004; Loizou et al., 2008). The underlying assumption of local analysis is that the model parameters are independent of each other, an assumption that is unlikely when representing a well-integrated physiological system. Moreover, interpretations of the local sensitivity analysis beyond the range of observed parameter values assume linear dependence on model output, which may not be accurate.

Global sensitivity analysis addresses the limitations of the local sensitivity analysis by simultaneously examining the model sensitivity over the entire range of uncertainty for all parameters. It also accounts for model non-linearities and parameter interaction effects within the defined ranges of parameter uncertainty. Previously we have summarized the need and utility of global sensitivity analysis in the field of predictive toxicology, and have conceptualized a workflow for global sensitivity analysis and demonstrated its use for PBPK models in adult humans (McNally et al., 2011, 2012). Here we present the implementation of a quantitative global sensitivity analysis of the BBDR-HPT axis pregnancy model for normal thyroid conditions at near-term, using an adaptation of the workflow developed in McNally et al. (2011). The methodological adaptations address the challenges in the feasibility of conducting such analyses for complex kinetic and dynamic models that are computationally demanding. Global sensitivity analysis of the BBDR-HPT axis pregnancy model allows for the ranking of the most influential and least influential model input parameters in addition to the characterization of their overall interaction and pair-wise interaction effects on the model output. Such systematic analyses help evaluate model behavior over a wide range of input conditions that are experimentally untested. It also aids in the future development of a probabilistic framework for the BBDR-HPT axis pregnancy model by reducing significantly the number of parameter evaluations and by increasing the confidence in the predictive potential of such models.

## Materials and methods

### Deterministic BBDR-HPT axis pregnancy model

**Figure 1:**
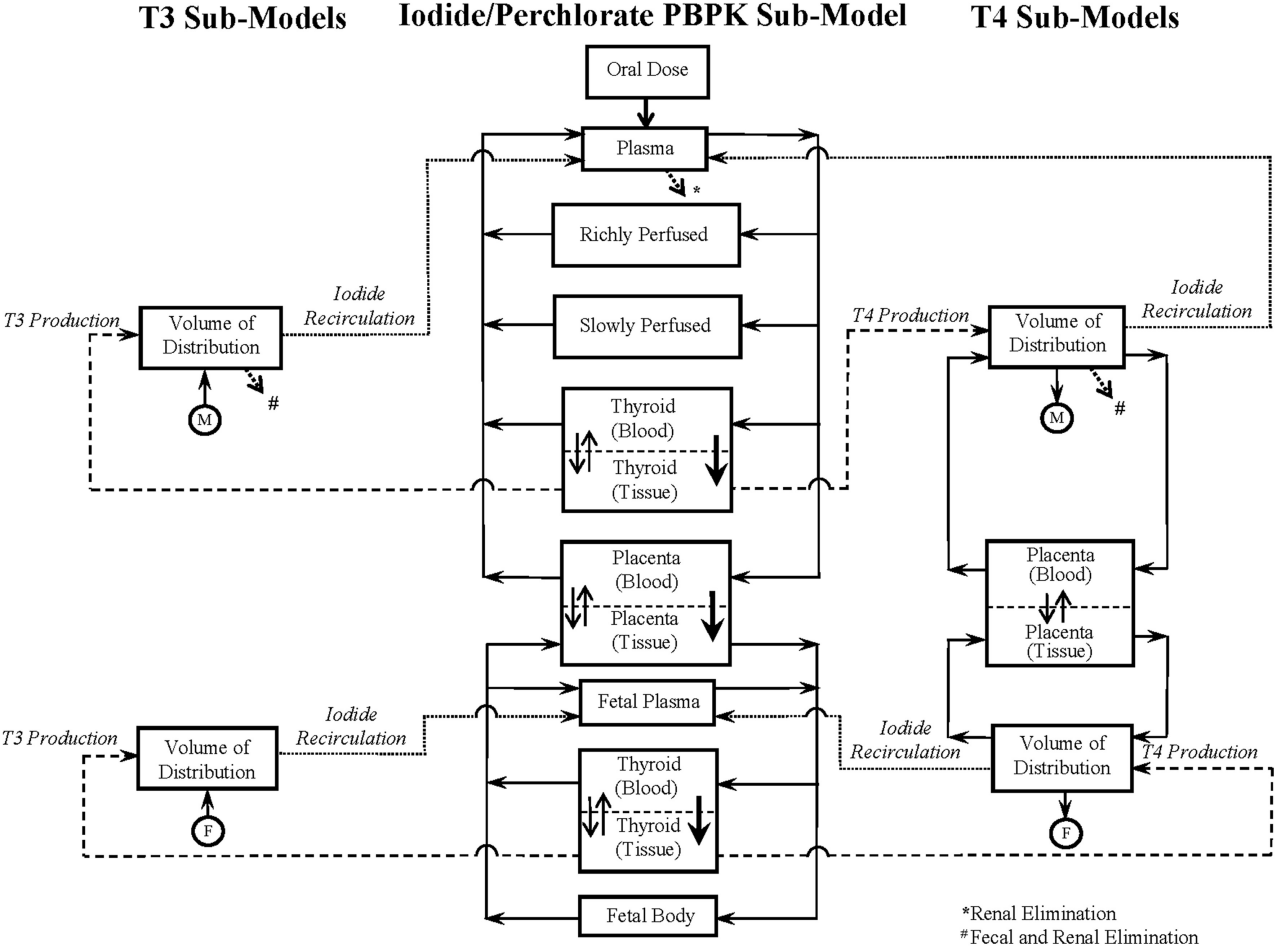
**BBDR-HPT axis model schematics for the near-term mother and fetus including the iodide PBPK submodel and thyroid hormone submodels for T4 and T3**. Following an oral intake dose, solid arrows with closed arrow heads 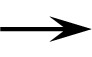 connecting the individual compartments in the anion PBPK submodel and the T4 volume of distribution represent the blood flows. Thicker arrows with closed arrow heads 
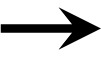
 and thinner open arrows 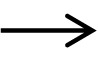 within the compartments denote the NIS mediated active uptake and bidirectional passive diffusion of anions across the thyroidal and placental sub-compartments, respectively. The dashed and dotted lines show the link between the PBPK submodels and the T4 and T3 submodels in both the mother and fetus. Dashed lines leaving the thyroid tissue of the iodide PBPK submodel in both maternal and fetal compartments into the hormone volume of distribution denote hormone production. Dotted lines leaving the hormone volume of distribution denote the recirculation of inorganic iodide released due to hormone metabolism into the PBPK submodel for iodide. The connector symbols from the maternal 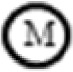 and fetal 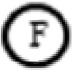 volume of distribution for T4 and T3 with solid closed arrows 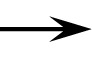 represents the metabolism of T4 to T3. The dotted open arrows 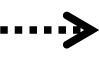 in the PBPK submodels and the thyroid hormone submodels represent the urinary (*) and combined urinary and fecal (#) elimination of iodide and thyroid hormones, respectively. PBPK submodel for perchlorate is similar to that of the iodide submodel excluding the organification in the thyroid and the subsequent links to the hormone volume of distributions. Perchlorate and iodide PBPK submodels are connected based on the mode of action of perchlorate and iodide to competitively inhibit each other at the sodium iodide symporter. Figure is taken with permission, is view only and permission must be obtained for any onward reuse (Lumen et al., 2013).

The schematic of the BBDR-HPT axis pregnancy model developed in Lumen et al. (2013) is shown in Figure 1. The comprehensive model includes a PBPK submodel for iodide and simple pharmacokinetic submodels for thyroid hormone, thyroxine (T4) and tri-iodothyronine (T3) in the mother and fetus. The maternal PBPK compartments for the anions include plasma, thyroid, placenta, and lumped rapidly and lumped slowly perfused compartments, whereas for the fetus, the body is described as one combined compartment, with separate plasma and thyroid subcompartments. Thyroid hormone submodels for T4 and T3 in the mother and fetus are described as simple volume of distributions. Maternal and fetal physiological parameters, such as tissue volumes, volume of distributions, and blood flows, are pre-defined in the model and are scaled to body weight and cardiac output. Chemical specific parameters, including partition coefficients, permeability area cross product terms, clearance rates, and fractional conversion terms, are used to describe the tissue distribution of anions and thyroid hormones. The PBPK submodel for perchlorate shares a similar model structure to the 8-compartment PBPK submodel for dietary iodide in the pregnant mother and fetus as represented in Figure 1.

The integrated BBDR model is structured and parameterized to describe the whole body disposition kinetics of dietary iodide. The model includes the sequestration of iodide into the thyroid via secondary active (sodium iodide symporter, NIS) and passive transport, followed by the intra-thyroidal organification and production of T4 and T3, the extra-thyroidal de-iodination of T4 to T3 and reverse T3 (rT3). The inorganic iodides released by de-iodination of T4, T3, and rT3 are described to circulate back into the pharmacokinetic component of the model contributing to the systemic iodide pool. The BBDR model accounts for the placental transfer of iodide both in its inorganic form and in the form of thyroxine during late gestation. First order kinetics is used in the deterministic model to describe the individual rate processes, such as modeling the thyroid function following the uptake of anions, and also the renal and fecal elimination processes of the inorganic and organic forms of iodide.

The iodide and thyroid hormone aspects of the model consists of 66 parameters and predicts the serum concentrations of maternal and fetal thyroid hormones at steady state including, total T4, free T4 and total T3 for various iodide intake conditions. Over 80 parameters are involved when the model is expanded to include the perchlorate submodel. In this current work we focus on the iodide kinetics and thyroid function descriptive submodels in the mother and fetus. The BBDR-HPT axis pregnancy model was calibrated over a range of dietary iodide intake for pregnancy, from 75 to 250 μg/day, where TSH is assumed not to be stimulated beyond its reference intervals. The calibrated model predicted serum and urinary iodide levels that were in concordance with observations and thyroid hormone levels that were within trimester-specific reference ranges. In the functional range of model evaluation for iodide intake, the model behavior is described to be non-linear. Urinary clearance rates of iodide were calibrated for a given iodide intake condition to predict the measured non-linear profile of thyroidal iodide stores with depleting iodide nutritional status. Physiological and chemical specific adaptive responses to alterations in thyroid hormone homeostasis and the maintenance of the serum levels of active hormone T3 were also accounted for in the model by iterative calibration of the de-iodination rate of T3 for varying iodide intake conditions. Additional information on the BBDR-HPT axis pregnancy model can be found in Lumen et al. (2013).

### Quantitative global sensitivity analysis methods and workflow

McNally et al. (2011) proposed a two-step approach to implement global sensitivity analysis for physiologically based pharmacokinetic models to reduce the computational burden. The workflow began with preliminary screening using the Morris method to eliminate the parameters with a negligible effect on the model output. Quantitative global sensitivity analysis of the selected subset of model parameters comprised the second-step of the workflow and was performed using the extended Fourier amplitude sensitivity test (eFAST) (McNally et al., 2011). The eFAST technique is a variance-based global method that is independent of any assumptions regarding model structure. eFAST provides an estimate of the variance of the chosen model output and the contribution of input parameters and their interactions to this variance, given physiologically feasible parameter ranges for inputs. The output of such an analysis are the main effect and total effect sensitivity indices. The main effect sensitivity index for a given parameter is the expected reduction in output variance if the parameter is known. It should be noted that, in general, summing the main effects across parameters may not total 100% because the main effects only contribute a portion of the variance and do not account for interaction effects. The total effect variance represents the expected amount of output variance that would remain unexplained (residual variance) if only that variable were left free to vary over its range, the value of all other variables being known. Total sensitivity indices are generally used to identify non-essential variables (i.e., those that have no importance either singularly or in combination with others). Large values of total effect relative to main effect imply the presence of interactions among model inputs. McNally et al. (2011) reported that sensitivity analysis of their relatively simple PBPK model using eFAST for 19 varying parameters took 13 h to execute. A single evaluation of this PBPK model took a fraction of a second.

The Morris method is particularly well suited for models such as the one evaluated in this current study with a large number of input parameters. However, eFAST was considered to be impractical due to the computational expense (a single evaluation of the BBDR-HPT axis pregnancy model took approximately 6½ min). A more complex, but vastly more efficient method of quantitative global sensitivity analysis was adopted in the present work. This involved the construction of a surrogate model, referred to in the literature as an emulator, and performing sensitivity analysis on the emulator. A workflow for the global sensitivity analysis of consequence models using an emulator has been described in Gant et al. (2013); a similar process has been adopted in our work.

In this study, Morris screening and quantitative global sensitivity analysis of the BBDR near-term pregnancy model for the thyroid axis was conducted at euthyroid conditions, with no perchlorate exposure and a dietary iodide intake dose of 200 μg/day (Lumen et al., 2013). In Lumen et al. (2013), model parameterizations and calibrations for the BBDR pregnancy model were conducted at steady state, where periodicity in model predictions for maternal serum thyroid hormone levels was achieved. Thus, the parameter screening using the Morris method was conducted over a period where steady state is expected to be achieved (2800–3000 h) and evaluating over a period of 200 h within the model's functional window of periodicity. Alterations in maternal serum fT4 levels were used as the relevant end point for disease state evaluations in Lumen et al. (2013) and are also chosen as the model predicted output for evaluation in the current work.

#### Parameter ranges

A summary of the model parameters (physiological and biochemical) and their ranges for input for the screening analysis is listed in Table 1. Point estimates of model parameters obtained from the literature and estimated in the deterministic model were used as the mean value of the distribution. The lower and upper bounds of the individual parameters were set to 2.5th and 97.5th percentile of a Gaussian distribution with parameter-specific variability, unless otherwise noted. A comprehensive literature search was conducted to survey the available information on variability and uncertainty around the mean for a given model parameter. Available information for life-stage-, thyroid axis system-, and chemical- specific model parameter variability was gathered and tabulated as shown in Table 1. When multiple studies characterizing variability were available for a given parameter, the data were combined using methodology adopted by Abduljalil et al. (2012). Where necessary, data were extracted from figures in original references using digitizing software. Coefficients of variation were calculated using values derived from literature searches and they are expressed for each as the ratio of standard deviation to the mean. Where information regarding variability in thyroid function is scarce for the near-term gestational time point being modeled, comparable information gathered from published studies involving non-pregnant individuals was used as initial estimates in the analysis. Coefficients of variation were assumed to be 30% for parameters with no variability or uncertainty information, unless specified otherwise. For lumped compartments, model re-parameterization as described in Gelman et al. (1996) was adopted to prevent the sampling of unrealistic values and to ensure that mass balance is maintained in compartments and blood flows to tissues (Gelman et al., 1996).

**Table 1:** **Ranges of physiological and biochemical parameters of the BBDR-HPT axis model for near-term pregnancy**.

**Parameter description**	**Parameter abbreviation**	**Value^a^**	**LB**		**UB**		**CV^b^**	**References**
**PHYSIOLOGICAL PARAMETERS (MOTHER AND FETUS)**								
Fetal body weight (kg)	BW_F	3.4	2.5		4.3		0.13	Abduljalil et al., 2012
Maternal body weight (kg)	BW_M	72.3	52.5		92.1		0.14	Abduljalil et al., 2012
Fetal cardiac output (L h^−1^ BW^−0.75^)	QFC_FI	32.6	14.8		50.4		0.28	Kenny et al., 1986; Kiserud et al., 2006
Maternal cardiac output (L h^−1^ BW^−0.75^)	QFC_MI	15.6	9.5		21.7		0.20	Clewell et al., 1999; Abduljalil et al., 2012
Blood flow to placenta (Percentage of maternal cardiac output)	QFPLC_MI	0.1	0.04		0.2		0.29	Abduljalil et al., 2012
Blood flow to fetal thyroid (Percentage of fetal cardiac output)	QFTHY_FI	0.016	0.01		0.03		0.30	–
Blood flow to maternal thyroid (Percentage of maternal cardiac output)	QFTHY_MI	0.016	0.01		0.03		0.34	Myant et al., 1949^c^
Volume of distribution for T3 in fetus (Proportion to fetal body weight)	VDFT3_FI	0.3	0.01		0.6		0.50	–
Volume of distribution for T3 in mother (Proportion to maternal body weight)	VDFT3_MI	0.46	0.2		0.7		0.24	Fisher and Oddie, 1964^c^; Nicoloff et al., 1972^c^
Volume of distribution for T4 in fetus (Proportion to fetal body weight)	VDFT4_FI	0.36	0.1		0.6		0.34	Oddie et al., 1966
Volume of distribution for T4 in mother (Proportion to maternal body weight)	VDFT4_MI	0.12	0.1		0.2		0.18	Dowling et al., 1967
Volume of placenta (Proportion to maternal body weight)	VFPLC_MI	0.009	0.01		0.01		0.16	Abduljalil et al., 2012
Volume of placental blood (Proportion to placenta mass)	VFPLCB_MI	0.233	0.1		0.3		0.19	Molteni et al., 1978
Volume of placental tissue (Proportion to placenta mass)	VFPLCT_MI	0.767	0.5		1.1		0.19	Molteni et al., 1978
Volume of fetal plasma (Proportion to fetal body weight)	VFPLS_FI	0.044	0.03		0.06		0.21	DeMarsh et al., 1942
Volume of maternal plasma (Proportion to maternal body weight)	VFPLS_MI	0.055	0.04		0.07		0.14	Abduljalil et al., 2012
Volume of fetal thyroid (Proportion to fetal body weight)	VFTHY_FI	3 × 10^−4^	1 × 10^−5^		6 × 10^−4^		0.50	Kay et al., 1966; Chanoine et al., 1991; van den Hove et al., 1999
Volume of maternal thyroid (Proportion to maternal body weight)	VFTHY_MI	2.35 × 10^−4^	1 × 10^−4^		4 × 10^−4^		0.57	Smyth et al., 1997
Volume of fetal thyroid blood (Proportion to fetal thyroid mass)	VFTHYB_FI	0.276	0.1		0.4		0.30	–
Volume of maternal thyroid blood (Proportion to maternal thyroid mass)	VFTHYB_MI	0.276	0.1		0.4		0.30	–
Volume of fetal thyroid tissue (Proportion to fetal thyroid mass)	VFTHYT_FI	0.724	0.3		1.1		0.30	–
Volume of maternal thyroid tissue (Proportion to maternal thyroid mass)	VFTHYT_MI	0.724	0.3		1.1		0.30	–
Maternal volume of urine (L)	VURINE	1.5	0.7		2.3		0.54	Thorp et al., 1999; Neithardt et al., 2002
**BIOCHEMICAL PARAMETERS (MOTHER AND FETUS)**								
Clearance rate for fetal intra-thyroidal binding (L h^−1^ BW^−0.75^)	CLF_BIND_FI	3000		750		5250	0.75	Dunning and Schwarz, 1981^c^,^d^
Clearance rate for maternal intra-thyroidal binding (L h^−1^ BW^−0.75^)	CLF_BIND_MI	3000		845		5155	0.37	Dunning and Schwarz, 1981^c^,^d^
Urinary clearance rate of iodide in mother (L h^−1^ BW^−0.75^)	CLF_UIM	0.17		0.1		0.3	0.32	Aboul-Khair et al., 1964
Elimination rate of T3 in mother (L h^−1^ BW^−0.75^)	CLFT3_MI	0.0027		0.0006		0.0048	0.77	Fisher and Oddie, 1964^c^; Habermann et al., 1978^c^
Elimination rate of T4 in mother (L h^−1^ BW^−0.75^)	CLFT4_MI	1.85 × 10^−4^		1 × 10^−4^		3 × 10^−4^	0.35	Fisher et al., 1971^c^; Habermann et al., 1978^c^
Fractional conversion term for T4 in fetus (no units)	FRCONVT4_FI	1.2 × 10^−4^		0.2 × 10^−4^		2.2 × 10^−4^	0.44	Greenberg et al., 1970; Erenberg et al., 1974; Oddie et al., 1977
Fractional conversion term for T4 in mother (no units)	FRCONVT4_MI	9 × 10^−5^		5 × 10^−5^		1.3 × 10^−4^	0.24	Skjoldebrand et al., 1982
Initial thyroidal iodide stores in fetus (mg)	IODSTORES_MG_FI	0.3		0.1		0.5	0.65	van den Hove et al., 1999
Initial thyroidal iodide stores in mother (mg)	IODSTORES_MG_MI	14.6		6.8		22.4	0.53	Delange, 1994^c^,^e^; van den Hove et al., 1999^c^,^e^; Zabala et al., 2009^c^
Degradation rate of T3 in fetus (1 h^−1^ BW^−0.75^)	KDEGT3F_FI	0.295		0.1		0.5	0.30	–
Degradation rate of T3 in mother (1 h^−1^ BW^−0.75^)	KDEGT3F_MI	0.002		0.001		0.003	0.30	–
Degradation rate of T4 in fetus (1 h^−1^ BW^−0.75^)	KDEGT4F_FI	0.004		0.002		0.005	0.22	Oddie et al., 1966
Degradation rate of T4 in mother (1 h^−1^ BW^−0.75^)	KDEGT4F_MI	1.9 × 10^−4^		1 × 10^−4^		3 × 10^−4^	0.30	–
Michaelis-Menten affinity constant for iodide and NIS (nM)	KMNIS_I	31500		8038.8		54961.2	0.38	Gluzman and Niepomniszcze, 1983; Kosugi et al., 1996
Production rate of T3 in fetus (1 h^−1^ BW^−0.75^)	KPRODT3F_FI	1.7 × 10^−5^		1 × 10^−5^		3 × 10^−5^	0.30	–
Production rate of T3 in mother (1 h^−1^ BW^−0.75^)	KPRODT3F_MI	2.2 × 10^−7^		4.4 × 10^−8^		4.0 × 10^−7^	0.41	Nicoloff et al., 1972^c^
Production rate of T4 in fetus (1 h^−1^ BW^−0.75^)	KPRODT4F_FI	1.7 × 10^−5^		0.7 × 10^−5^		2.7 × 10^−5^	0.30	–
Production rate of T4 in mother (1 h^−1^ BW^−0.75^)	KPRODT4F_MI	2.5 × 10^−6^		6.9 × 10^−7^		4.2 × 10^−6^	0.37	Nicoloff et al., 1972^c^
Permeability area cross product term for iodide in placenta (L h^−1^ BW^−0.75^)	PAFPLC_MI	0.005		0.002		0.008	0.62	Blount et al., 2009^f^
Permeability area cross product term for iodide from placenta blood to tissue (L h^−1^ BW^−0.75^)	PAFPLCBTOT_MI	0.08		0.03		0.1	0.62	Blount et al., 2009^f^
Permeability area cross product term for iodide from placenta tissue to blood (L h^−1^ BW^−0.75^)	PAFPLCTTOB_MI	0.08		0.03		0.1	0.62	Blount et al., 2009^f^
Permeability area cross product term for fT4 in placenta (L h^−1^ BW^−0.75^)	PAFT4PLCF_MI	2.8 × 10^−4^		1 × 10^−4^		4 × 10^−4^	0.60	Vulsma et al., 1989
Permeability area cross product term for iodide in fetal thyroid (L h^−1^ BW^−0.75^)	PAFTHY_FI	1 × 10^−4^		4 × 10^−5^		1.6 × 10^−4^	0.30	–
Permeability area cross product term for iodide in maternal thyroid (L h^−1^ BW^−0.75^)	PAFTHY_MI	1 × 10^−4^		4 × 10^−5^		1.6 × 10^−4^	0.30	–
Partition coefficient for fT4 in placenta (no units)	PFT4PLC_MI	1.44		0.6		2.3	0.30	–
Partition coefficient for iodide in placenta (no units)	PPLC_MI	0.4		0.2		0.6	0.30	–
Partition coefficient for iodide in cord blood (no units)	PPLCPF_MI	0.4		0.2		0.6	0.30	–
Partition coefficient for iodide in fetal rest of the body tissues (no units)	PROB_FI	0.4		0.2		0.6	0.30	–
Partition coefficient for iodide in maternal richly perfused tissues (no units)	PRP_MI	0.4		0.2		0.6	0.30	–
Partition coefficient for iodide in maternal slowly perfused tissues (no units)	PSP_MI	0.18		0.1		0.3	0.30	–
Partition coefficient for iodide in fetal thyroid (no units)	PTHY_FI	0.15		0.1		0.2	0.30	–
Partition coefficient for iodide in maternal thyroid (no units)	PTHY_MI	0.15		0.1		0.2	0.30	–
Maternal partition coefficient for TT4 in placenta (no units)	PTT4PLC_MI	1.44		0.6		2.3	0.30	–
Length of dietary exposure for iodide (h)	TLEN_I	1		0.4		1.6	0.30	–
Vmax for iodide and NIS in placenta (nmol h^−1^ BW^−0.75^)	VMAXNISF_PLC_MI	750		285		1215	0.62	Blount et al., 2009^f^
Vmax for iodide and NIS in fetal thyroid (nmol h^−1^ BW^−0.75^)	VMAXNISF_THY_FI	3900		975		6825	0.75	Dunning and Schwarz, 1981^c^,^d^
Vmax for iodide and NIS in maternal thyroid (nmol h^−1^ BW^−0.75^)	VMAXNISF_THY_MI	3800		478		7122	0.45	Pochin, 1952^d^; Halnan, 1958; Aboul-Khair et al., 1964

*LB, Lower Bound; UB, Upper Bound; CV, Coefficient of variation*.

a *Mean values represent the literature derived and model calibrated point estimates in Lumen et al. (2013)*.

b *Determination of coefficient of variations from available literature sources*.

c *Approximated from estimates derived from non-pregnant subjects*.

d *Assuming that the estimated 24-h radioiodide uptake is reflective partly or wholly of intra-thyroidal iodide binding and the active thyroidal clearance of iodide*.

e *Parameters for which estimates were combined from multiple studies*.

f *Estimates of variability and uncertainty around those estimates were derived from reported population estimates of matched cord blood and maternal serum iodide levels*.

#### The morris screening method

The Morris method, as described in McNally et al. (2011), was adapted for use as a screening test for the BBDR-HPT axis near-term pregnancy model parameters. Sixty-six maternal and fetal model input parameters, inclusive of physiological, iodide- and thyroid-hormone specific parameters, were screened. The model runs were allowed to reach periodicity for evaluating the predicted output of maternal fT4 levels. The interference factor and the number of re-samplings were set to 4 and 1, respectively, as recommended in Saltelli et al. (1999). In Morris screening, the influence of each parameter is assessed with two sensitivity measures: μ, which measures the overall influence of a variable and σ, which estimates the interaction propensity or non-linearity effect. Parameters were evaluated using an input space represented by 100 Morris optimized trajectories. A total of 100 elementary effects was sampled from the finite distributions of each parameter, from which μ and σ were derived (Campolongo et al., 2007). Due to the stochastic nature of the Morris method, the screening analysis in its entirety was repeated three times and the consistency in the parameter ranking was compared across runs. Results of the screening analysis were analyzed by ranking model parameters in descending order of μ and σ for the individual iterations. Results based on Morris screening were compared to those obtained from one-at-at-time local sensitivity analysis.

#### Gaussian process emulation

Model parameters selected by the Morris method were used as input for the quantitative global sensitivity analysis, which was performed using Gaussian Emulation Machine (GEM) software. In contrast to a method like eFAST, which can be executed in a single script file and with no user interaction or expertise required, the method used in our work requires some mathematical expertise and greater interaction with the user. A concise description of the process is provided here. However greater technical detail, sufficient to replicate our work, is provided in Supplementary Material. The efficiency of this technique arises from the use of an emulator, which is a statistical approximation to the BBDR model. The emulator used in this work was based upon a Gaussian Process regression model that has certain properties that are suitable for use with deterministic models (Oakley and O'Hagan, 2004; Kennedy, 2005; Gant et al., 2013). The emulator was built by using a relatively modest sample of model evaluations. The maximum and minimum values for each of the parameters were input into GEM and the software generated a 250 run maxi-min Latin hypercube design (Kennedy, 2005). Each run of the design corresponds to a unique set of parameters in the BBDR-HPT axis pregnancy model. The models were run and the output saved. The emulator was built using the input and output files. Once built, the emulator was highly efficient and could be used to approximate the model output (and quantify the uncertainty in this estimate) at untried inputs: this was computed in a fraction of a second compared with the 6½ min to run the BBDR-HPT axis pregnancy model itself. It was important to assess the quality of fit of the emulator, which was done by cross validation; this was essentially a comparison of known model outputs against the emulator predictions. If cross validation errors are small the emulator can be regarded as a reliable surrogate for the BBDR model (see Supplementary Material for greater technical detail). The output generated by GEM was used to assess the quality of fit of the emulator. This process of model evaluation suggested the ranges of some of the model parameters were too wide because the output from the BBDR-HPT axis pregnancy model was physiologically irrelevant at values close to the specified minimum and maximum values. The parameter ranges were revised until this unusual behavior was eliminated.

### Software

The BBDR-HPT axis pregnancy model was scripted and solved using advanced continuous simulation language (acslX) version 3.0 (The AEgis Technologies Group, Inc., Huntsville, AL). Data extraction from plots was performed using the graph digitizing software DigitizeIT 1.5 (Share It, Braunschweig, Germany). Model simulations were run on a Dell laptop computer equipped with Intel® Core™ i5-3210M @2.5GHz processors and Windows 7 operating system. Morris screening analysis was performed on a Dell workstation equipped with Intel® Xeon® CPU @3.47GHZ (2 processors) and Windows 7 operating system. Sets of 250 model runs were run as batch processes in parallel using the high performance scientific computing laboratory clusters at the FDA/Center for Devices and Radiological Health (CDRH)/Office of Science and Engineering Laboratories (OSEL). One cluster comprised 110 IBM System x3650 M2 8-core diskless compute nodes, whereas the other comprised 252 IBM iDataPlex dx360 M2 8-core diskless compute nodes. The run scripts for the Morris method were provided by Dr. George Loizou (Health and Safety Laboratory, Buxton, UK). GEM software v1.1 (http://www.tonyohagan.co.uk/academic/GEM/index.html) was used for quantitative global sensitivity analysis. Lowry plots were created in R using scripts provided in the appendix of McNally et al. (2011).

## Results

**Figure 2:**
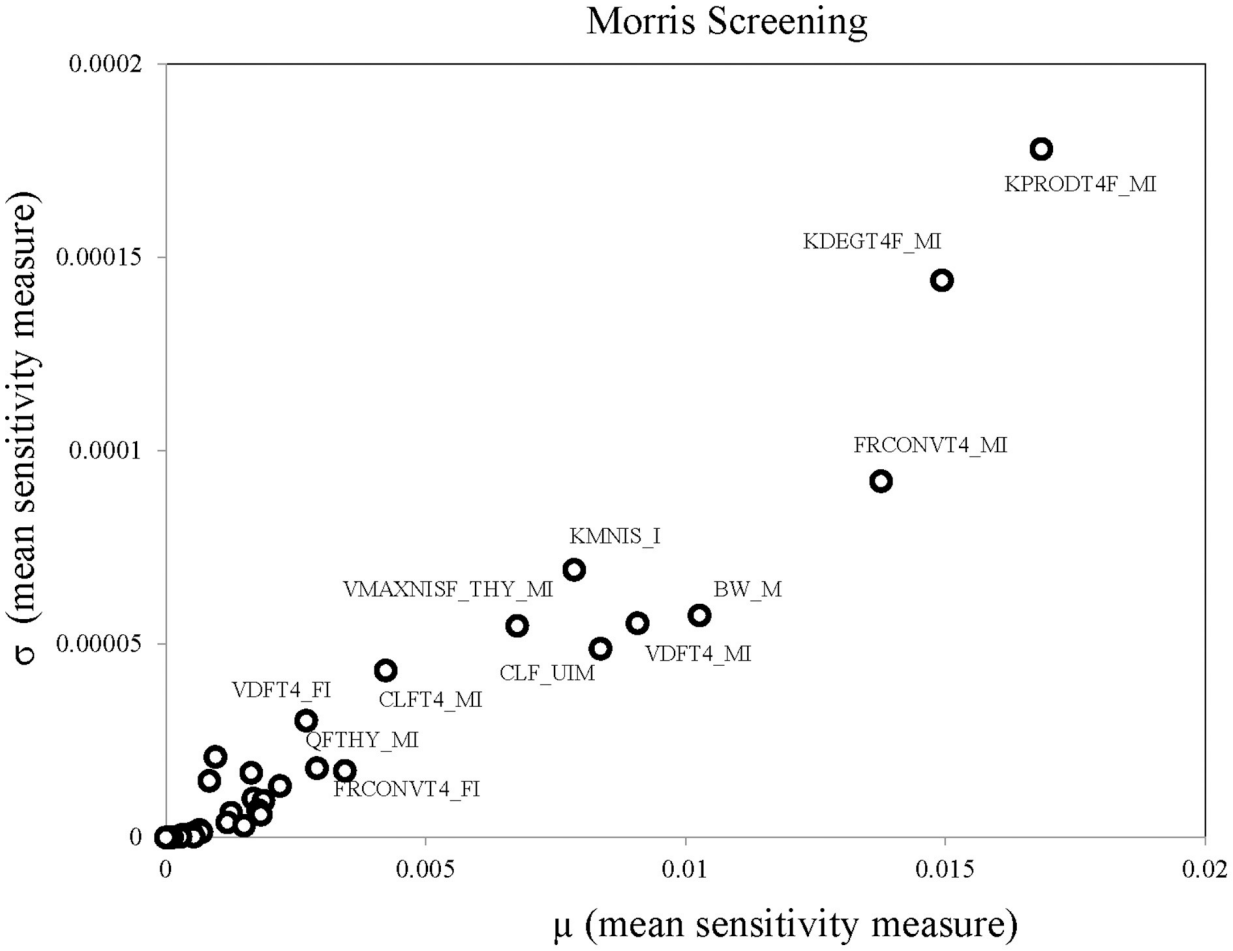
**Morris screening analysis results identifying the most influential parameters on model predicted output of maternal fT4 levels at steady state**. Mean sensitivity indices, μ and σ, for each model input parameter are as denoted.

Parameter ranges and the corresponding coefficient of variations for the 66 model parameters, as shown in Table 1, were input variables for the Morris screening analysis. Figure 2 shows a scatterplot of the two sensitivity indices μ and σ for a representative run. In this figure, parameters with relatively high μ and σ are labeled individually, as densely as legibility permits. The sensitivity indices μ and σ calculated for all three iterations of the Morris method screening analysis are presented in Supplementary Table 1. The input parameters of the euthyroid BBDR-HPT axis pregnancy model to predict maternal thyroid hormone levels were ranked by each sensitivity measure and compared across stochastic runs as shown in Supplementary Table 2. Based on the semi-quantitative criteria of having distinctively higher μ (overall influence of a variable) and higher σ (parameter interaction potential or non-linearity effect of a variable), a sub-set of 26 model parameters were screened for quantitative global sensitivity analysis with μ greater than 6e-4. The maternal thyroidal iodide store parameter was also included in the screened set of parameters for the verification of the influence of initial values of thyroidal iodide stores in the BBDR-HPT axis pregnancy model. Given the steady state nature of the deterministic model, the Morris screening test was run to a pre-defined time period where periodicity in maternal thyroid hormone levels is expected to be achieved. The computational run time for each Morris screening test of the model at steady state and for all 66 input parameters evaluated was approximately 72 h on a Dell computer workstation. A comparison of the parameter ranking based on the computed main effect or overall influence on model output by the Morris method and one-at-a-time local sensitivity analysis are tabulated in Table 2.

**Table 2:** **Comparison of parameter ranking results of Morris screening and Local Sensitivity Analysis**.

**Parameters**	**Morris screening**	**Local sensitivity analysis**
KPRODT4F_MI	1	17
KDEGT4F_MI	2	4
FRCONVT4_MI	3	3
BW_M	4	1
VDFT4_MI	5	5
CLF_UIM	6	2
KMNIS_I	7	6
VMAXNISF_THY_MI	8	7
CLFT4_MI	9	12
FRCONVT4_FI	10	13
QFTHY_MI	11	10
VDFT4_FI	12	14
PFT4PLC_MI	13	18
VMAXNISF_THY_FI	14	23
PAFPLCTTOB_MI	15	21
PPLCPF_MI	16	22
PPLC_MI	17	20
PAFT4PLCF_MI	18	25
QFC_MI	19	11
KDEGT4F_FI	20	15
PAFPLCBTOT_MI	21	24
KPRODT4F_FI	22	35
QFC_FI	23	32
QFRP_MI	24	8
QFTHY_FI	25	31
VMAXNISF_PLC_MI	26	27
KPRODT3F_MI	27	16
BW_F	28	19
QFROB_FI	29	50
QFSP_MI	30	9
QFPLC_MI	31	39
IODSTORES_MG_FI	32	60
KDEGT3F_MI	33	29
CLFT3_MI	34	28
KPRODT3F_FI	35	34
VFRP_MI	36	38
VDFT3_MI	37	30
VFPLC_MI	38	42
VFPLCT_MI	39	26
VFSP_MI	40	37
PRP_MI	41	40
PAFTHY_MI	42	41
VFPLCB_MI	43	33
PSP_MI	44	36
PROB_FI	45	46
VFPLS_MI	46	44
TLEN_I	47	43
PAFTHY_FI	48	47
KDEGT3F_FI	49	57
VFPLS_FI	50	51
VFROB_FI	51	45
VFTHY_MI	52	48
VFTHYB_MI	53	49
VFTHY_FI	54	54
CLF_BIND_MI	55	52
VFTHYB_FI	56	55
PTHY_MI	57	53
CLF_BIND_FI	58	58
PTHY_FI	59	59
VFTHYT_FI	60	62
VFTHYT_MI	61	61
IODSTORES_MG_MI	62	56
VDFT3_FI	63	65
VURINE	64	66
PAFPLC_MI	65	63
PTT4PLC_MI	66	64

**Figure 3:**
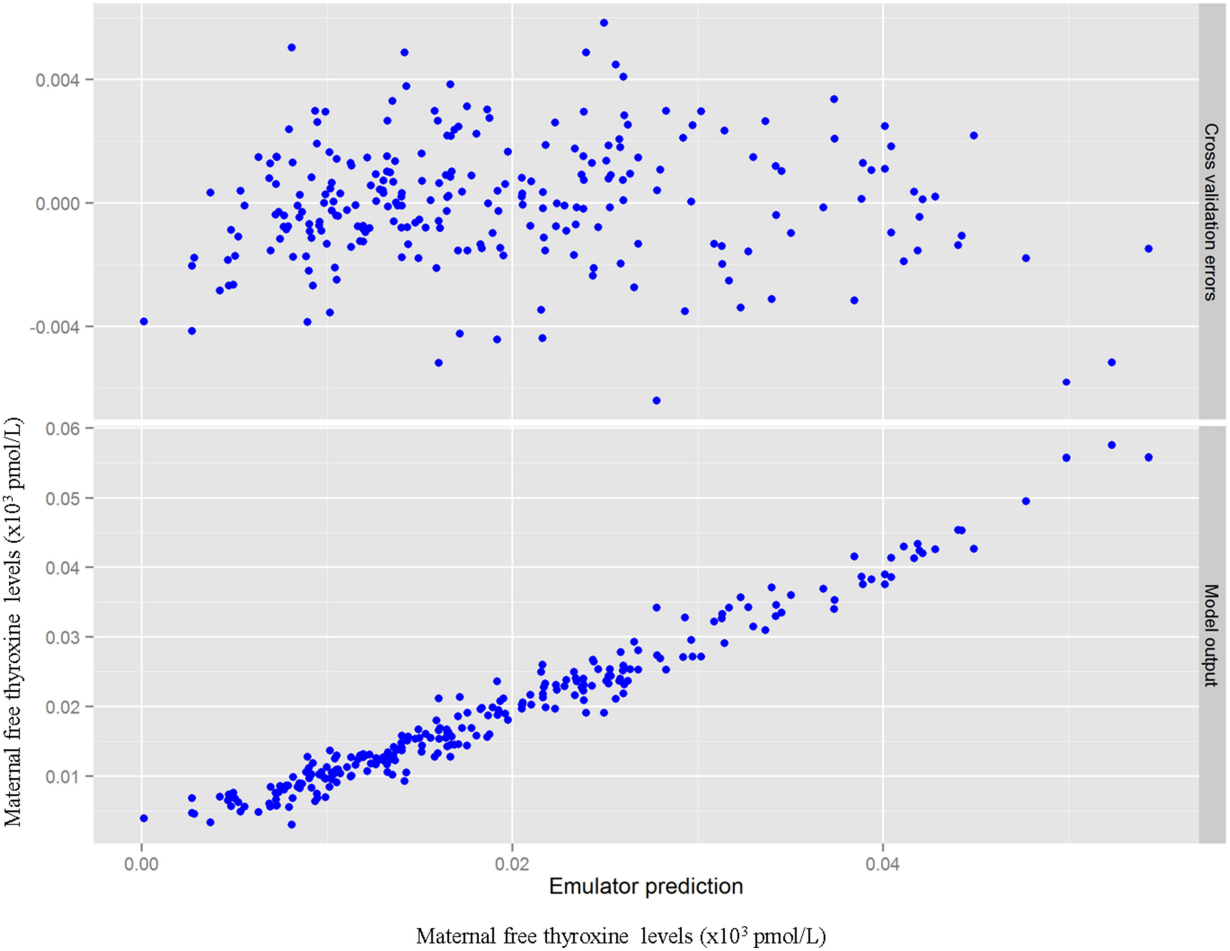
**Cross validation errors and predictions from the emulator plotted against the BBDR-HPT axis pregnancy model output of maternal free thyroxine levels (pmol/L)**.

**Figure 4:**
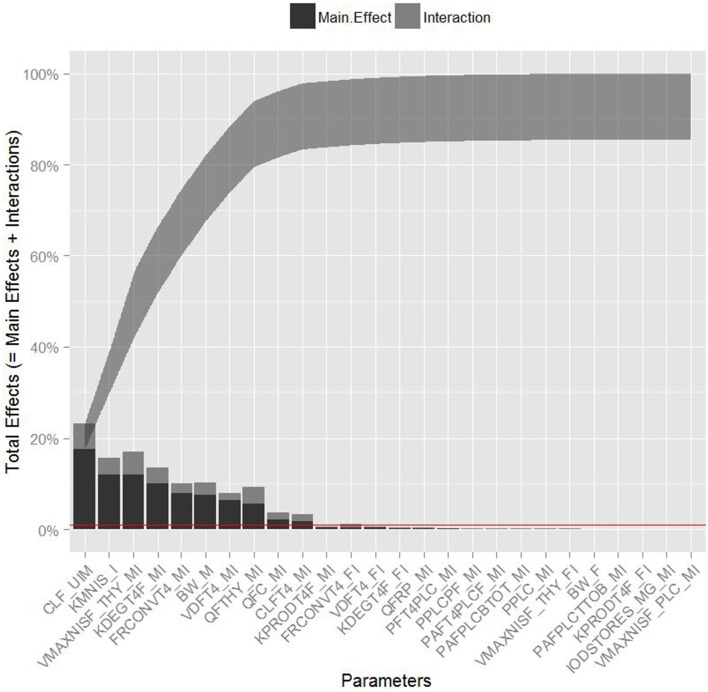
**Lowry plot of the quantitative global sensitivity analysis results of Gaussian Emulation processes**. The total effect of a parameter comprised the main effect (black bar) and any interactions with other parameters (gray bar) given as a proportion of variance. The ribbon, representing variance due to parameter interactions, is bounded by the cumulative sum of main effects and the minimum of the cumulative sum of the total effects for model predicted levels of maternal free thyroxine levels at steady state. Red line denotes the model parameters with variances and/or total effect greater than 0.5%.

**Figure 5:**
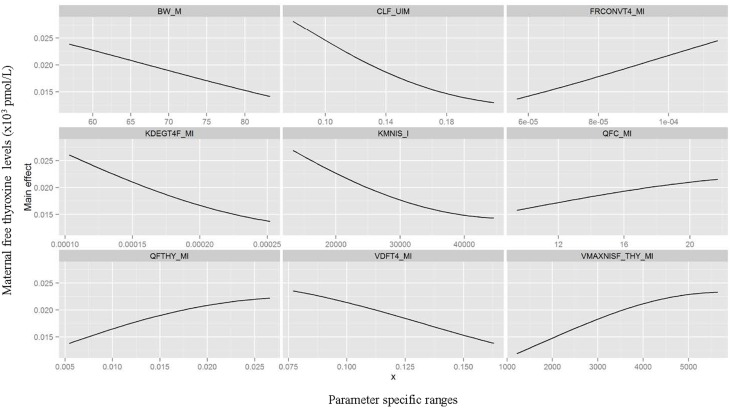
**Gaussian Emulation process outputs**. Trend plots of the main effects on model predicted output for the nine parameters identified as most influential varied over its simulation range from minimum to maximum value.

Table 3 summarizes the domain of the screened input parameters and numerical results of the global sensitivity analysis using GEM. Prior to interpreting the sensitivity indices produced by GEM it is important to assess the quality of fit of the emulator. Unless the emulator is an adequate surrogate for the BBDR-HPT axis pregnancy model, the sensitivity analysis based upon the emulator might be unreliable. Accuracy of the emulator was assessed by an analysis of cross validation errors. Checks are analogous to those on the residuals from a multiple regression model: the predictions and model outputs should lie on a 1-to-1 line (i.e., the emulator is unbiased) with errors that show no discernable trend with model output or any model inputs. Figure 3 demonstrates that the emulator was a reasonable fit to the simulation results. There was an indication that the emulator may have a small degree of bias, spanning the range of the deterministic model calibration, for maternal free thyroid hormone levels that are close to zero. This bias should only have a minor impact on the sensitivity indices; however, it does identify the unique behavior of the BBDR model at maternal thyroid hormone levels that are close to zero. Model parameters with variances and/or total effect greater than 0.5% were noted to be influential variables and were thus identified as the best subset of model input parameters. The contributions of main and total effects for each parameter are illustrated in a Lowry plot (Figure 4). The vertical bars depict the main and the total effects of each of the parameter, ranked in descending order of main effect. The ribbon on the top is a confidence band for the cumulative sum of model output variance. The analysis shows that the top 9–11 parameters contribute 80–100% of the output variance, suggesting that there is some indication of higher order interactions. Plots of the main effects, which show the trend in maternal thyroid hormone levels if the parameter is varied over its simulation range from minimum to maximum values, are shown for the nine most important parameters in Figure 5. These are an additional output available from GEM that alternative variance-based methods cannot compute.

**Table 3:** **Global sensitivity analysis parameter inputs and quantitative output indices**.

**Parameters**	**Lower bound**	**Upper bound**	**Variance (%)**	**Total effect**
CLF_UIM^*^	7.87E-02	2.11E-01	17.60	23.00
KMNIS_I^*^	1.33E+04	4.46E+04	12.00	15.70
VMAXNISF_THY_MI^*^	1.22E+03	5.64E+03	12.00	17.00
KDEGT4F_MI^*^	1.03E-04	2.52E-04	10.20	13.60
FRCONVT4_MI^*^	5.67E-05	1.14E-04	8.03	10.10
BW_M^*^	5.69E+01	8.33E+01	7.57	10.20
VDFT4_MI	7.69E-02	1.63E-01	6.45	8.05
QFTHY_MI	5.43E-03	2.66E-02	5.58	9.23
QFC_MI	9.48E+00	2.17E+01	2.15	3.76
CLFT4_MI	5.66E-05	3.13E-04	1.77	3.34
KPRODT4F_MI	6.85E-07	4.21E-06	0.44	0.67
FRCONVT4_FI	1.56E-05	2.24E-04	0.43	1.24
VDFT4_FI	1.21E-01	5.99E-01	0.33	0.64
KDEGT4F_FI	2.13E-03	5.47E-03	0.24	0.42
QFRP_MI	4.51E-01	8.37E-01	0.18	0.31
PFT4PLC_MI	5.93E-01	2.29E+00	0.14	0.25
PPLCPF_MI	1.65E-01	6.35E-01	0.09	0.25
PAFT4PLCF_MI	1.12E-04	4.48E-04	0.08	0.19
PAFPLCBTOT_MI	3.04E-02	1.30E-01	0.06	0.13
PPLC_MI	1.65E-01	6.35E-01	0.06	0.12
VMAXNISF_THY_FI	9.75E+02	6.82E+03	0.06	0.13
BW_F	2.53E+00	4.27E+00	0.03	0.09
PAFPLCTTOB_MI	3.04E-02	1.30E-01	0.02	0.04
KPRODT4F_FI	8.24E-05	3.18E-04	0.00	0.00
IODSTORES_MG_MI	6.81E+00	2.24E+01	0.00	0.00
VMAXNISF_PLC_MI	2.85E+02	1.22E+03	0.00	0.00

* *Parameter ranges optimized for physiologically plausible sampling space for global sensitivity analysis*.

Maternal urinary clearance rate of iodide ranks highest in the estimates of main and total effect and shows a negative correlation with maternal thyroid hormone levels (Figure 5). The model parameters of the following three ranks have similar main and total effect contributions. Two of the three parameters are Michaelis-Menten enzyme kinetic parameters, Km (affinity constant of iodide) and Vmax (maximal reaction rate of thyroidal iodide uptake) of the sodium iodide symporter in the thyroid, with negative and positive correlations with maternal thyroid hormone levels, respectively. The third parameter is a thyroid hormone-specific parameter which is a first order de-iodination rate constant of maternal thyroxine determining the release of inorganic iodide from its organic forms and shows negative correlations with maternal thyroid hormone levels. The subsequent parameters contribute less than 10% to total output variance.

Urinary clearance rate of iodide, the Michaelis-Menten kinetic parameters, and the blood flow to the maternal thyroid show the highest parameter interaction quotients. GEM also allows the calculation of specific two-way parameter interaction variances. The interactions accounting for greater than 0.5% of variance were between affinity constant of iodide to NIS and the maternal urinary clearance rate of iodide; affinity constant of iodide to NIS and blood flow to the maternal thyroid; maximal reaction rate of NIS and the maternal urinary clearance rate of iodide; and maximal reaction rate of NIS and blood flow to the maternal thyroid. Lack of fit of the emulator (in particular for predictions close to zero) can account for some of variance apportioned to interactions; therefore, the weaker interactions should be viewed with some caution.

## Discussion

The biologically based dose-response model for the hypothalamus pituitary thyroid axis constitutes a complex network of anion kinetic submodels and thyroid hormone specific submodels. The model is first of its kind to describe the disposition of iodide following dietary intake and to capture its subsequent biosynthesis and secretion of thyroid hormones in the mother and fetus for near-term pregnancy conditions. A considerable yet identifiable set of physiological-, iodide- and thyroid hormone-specific parameters were used to configure the model structure for whole body iodide kinetics and thyroid function (Lumen et al., 2013). Global sensitivity analysis as opposed to one-at-a-time local sensitivity analysis, are capable of investigating the nature of parameter influence and quantifying their individual and interaction effects on a chosen model output. Maternal thyroid hormones play a crucial role in fetal neurodevelopment, hence perturbation of serum levels of maternal fT4 was chosen as the relevant endpoint and model output for analysis.

Parameter evaluation ranges were derived for each model input parameter as available in the literature and reasonable assumptions were made on likely ranges where data were not available. Where appropriate, a meta-analysis was conducted to derive a pooled estimate of the variabilities and uncertainties on parameters with datasets from multiple studies. The point estimates of the parameters calibrated in the deterministic model served as the parameter means around which the deduced distributions were developed. The coefficient of variation was used to quantify dispersion for each variable and the lower and upper limit of expression captured 95% of the Gaussian distribution. These distributions for each of the 66 model input parameters were inputs to the Morris Screening analysis of the model at steady state. Qualitative ranking of model parameters following the Morris screening analysis was reasonably consistent among stochastic iterations (McNally et al., 2011). Of the 66 input parameters, the 26 that had distinguishably higher sensitivity indices were screened for quantitative global sensitivity analysis. These 26 parameters included mostly of maternal iodide and thyroid hormone specific parameters with few physiological and fetal parameters. In addition, the robustness of the overall screening analysis was noted when consistent results were obtained when the analysis was repeated for alternate representative time intervals for steady state and lower bound dietary iodide intake level of 75 μg/day (simulations not shown). As expected, a considerable degree of concordance based on the main effect can be noted amidst the top-ranked and bottom-ranked parameters computed by the Morris screening analysis compared to that of the local sensitivity analysis.

Quantitative global sensitivity analysis is computationally expensive requiring many thousands of model evaluations. Computational cost and efficiency plays a crucial role in decisions regarding modeling approaches for global sensitivity analysis, particularly for complex systems with a large number of parameters and a long runtime for each simulation (McNally et al., 2011; Gant et al., 2013). An emulator based upon a Gaussian Process regression model was chosen over eFAST primarily due to computational efficiency: eFAST was not computationally feasible. The emulator also provides richer model output compared with eFAST in the form of main effect plots and the computation of specific two-way interactions (Gant et al., 2013). However, there is an additional tier of complexity in assessing the fit of the emulator, and the methodology requires greater user engagement in generating the Latin Hypercube design, running the simulations, and then reading in input and output data to perform the sensitivity analysis. The Latin Hypercube design used in this work was particularly useful since it allowed the efficient sampling of high dimensional parameter space. We were thus able to quantify the range of maternal thyroid hormone levels predicted by the BBDR-HPT axis pregnancy model that were consistent with outputs as expected for input parameter ranges specified. In the modeling process we learned that some input ranges were too large because they corresponded to physiologically unrealistic maternal thyroid hormone levels and the parameter ranges were subsequently reduced. For the 26 screened parameters, analysis of the 250 BBDR pregnancy model runs using the GEM software had a run time of less than 40 min. While the screening analysis of 60+ parameters using the Morris method at steady state was computationally expensive, this step was critical in identifying the most influential parameters and achieving computational efficiency in the global sensitivity analysis phase.

In summary, global sensitivity analysis quantified 11 parameters with main and total effects higher than 0.5%. Interestingly, the Gaussian emulation process ranked maternal iodide-specific parameters equally as high as maternal thyroid hormone-specific parameters in their contribution to the total variance of the model predicted maternal thyroid hormone levels. The iodide-specific parameters with relatively high sensitivity indices include maternal urinary clearance rate of iodide and the Michaelis-Menten enzyme kinetic parameters, Km and Vmax of the sodium iodide symporter and the blood flow to the maternal thyroid. The thyroid hormone related parameters include maternal thyroxine production rate, degradation rate, urinary clearance rate, thyroxine volume of distribution, and the fractional conversion factor that determines the unbound free fraction of T4 in the serum. These identified sensitive model parameters support the conceptual framework underpinning the factors that affect iodide kinetics and thyroid function, and hence the maternal thyroid hormone levels. Although ranked lower, a few of the fetal thyroid hormone-specific parameters were also determined to be influential, such as the volume of distribution of thyroxine in the fetus and the free fraction of fetal thyroxine as determined by the thyroid hormone binding proteins in the fetus.

The three highest ranking parameters identified by global sensitivity analysis include the urinary clearance rate of iodide and the Michaelis-Menten parameters, Km and Vmax that determine the sodium iodide symporter mediated thyroidal uptake of iodide. The main effects of these three variables and their pair-wise interactions as identified by the global sensitivity analysis succinctly summarize the interplay between the two kinetically competing mechanisms, renal elimination of iodide and sequestration into the thyroid, that influence the circulating levels of inorganic iodide in the pregnant woman. Inorganic iodide is the main and limiting substrate for thyroid hormone biosynthesis. The thyroid hormone-specific parameters identified as influential, such as thyroxine degradation rate, volume of distribution, and the fractional conversion term for serum thyroid hormone and protein binding, can also directly modulate the serum concentrations of the maternal fT4 levels. Intra-thyroidal production rate of thyroxine was also captured as a sensitive parameter but was ranked lower compared to the aforementioned parameters.

Identification of blood flow to maternal thyroid as a sensitive parameter in addition to the deduced parameter interaction coefficients with the sodium iodide symporter parameters (Km and Vmax) emphasizes the flow-limited nature of the system and the importance of local availability of iodide as substrate for thyroidal uptake. In addition to blood flow, maternal body weight, to which several model parameters are scaled, was not surprisingly identified as a crucial physiological parameter contributing to model output variance. Collectively these results indicate that the maternal intra-thyroidal iodide stores play a pivotal role in linking the inorganic iodide substrate kinetics of NIS mediated thyroidal uptake of iodide to the dynamic aspects of thyroid hormone production and alterations in serum thyroid hormone levels at steady state. It can be noted that the sustenance of the thyroidal iodide stores is the rate limiting step in the thyroid hormone production and not the rate of thyroxine production itself. The findings of this analysis highlight the need for future studies focusing on determining intra-thyroidal iodide stores and serum inorganic iodide levels in sensitive sub-populations such as pregnant mother and fetus to provide information on iodide nutritional and thyroid function status, in addition to the common biomarker measurements such as urinary iodide and serum thyroid hormone levels. Although the contribution of parameter interactions to total output variance is lesser than its main effects, identification of the interacting parameters aided in the better understanding of the true descriptors of the thyroidal system and verifies the models ability to emulate the functional aspects of the biological system under study, in these sensitive life-stages. Moreover, the systematic identification of influential model parameters due to not only their main effects but by interactions as in the case of the urinary clearance rate of iodide and blood flow to the thyroid among others, stresses the potential use of global sensitivity analysis in prioritizing future research through a holistic system evaluation approach.

Additional study outcomes including parameter in-sensitivity may help inform and refine our understanding of the model and of the modeled system. For example, as discussed above the thyroidal iodide stores are found to play a crucial role in this dynamic system but the importance of its parameter initialization is found to be minimal. Other relatively lower-ranked maternal parameters include tissue partition coefficients of fT4 that might influence the circulating levels of serum thyroid hormone levels. Parameters that drive the placental transfer between the mother and the fetus, such as permeability area cross product terms and enzyme kinetic parameters for both fT4 and iodide, were screened and identified to be influential but to a lesser degree. Post-calibration estimates in the deterministic model suggest that 20% of the fetal thyroxine levels are maternal in origin via trans-placental contributions, with the remaining 80% due to fetal intra-thyroidal production at near term. Parameters such as volume of distribution and degradation rate of fetal thyroxine, fractional conversion terms between total and free fetal thyroxine, and sodium iodide symporter mediated thyroidal iodide uptake parameters in the fetal thyroid were all identified as sensitive parameters but are ranked lower in comparison to equivalent parameters in the mother. These findings suggest that the fetal submodel structure taking into account its functionality does not completely act as a sink to the maternal submodel as the fetal thyroid develops in utero.

This study offers a good example of the application of quantitative global sensitivity analysis for a BBDR-HPT axis pregnancy model and its use in the understanding of the fundamental principles of an intricate biological system, stressing the importance of the work in its methodological and science-based applications. The study also helped identify sources of uncertainties in the BBDR-HPT axis model output and quantify their contributions as main, total, and interaction effects. The Lowry plot offers a visually comprehensible means to present the outcomes of the global sensitivity analysis for such complex models, facilitating communications between model developers and application-orientated researchers (McNally et al., 2011). The knowledge gained in this study sets the stage for conducting a computationally efficient probabilistic analysis of the BBDR-HPT axis pregnancy model using the identified sub-set of model input parameters with their contributions to model uncertainties quantified. Whilst the methodology for the global sensitivity analysis utilized in this work was developed in 2004 and has been applied to a diverse range of models, there are relatively few applications of the methodology outside specialist literature on statistics and computer models (Oakley and O'Hagan, 2004). To our knowledge this is the first application of global sensitivity analysis based upon an emulator in the toxicology/pharmacology literature. More than ever in recent times where the acceptance and application of computational models are rapidly increasing, it is of importance to continue investing in the science of modeling and current practices as needed in support of its advancement. The demonstration of the computational feasibility of quantitative global sensitivity analysis for larger biological models using cross-disciplinary methodologies as shown in this work provides current and future modelers with a workflow for use and application as they deem fit. The impact of such modeling practices extends beyond its use in the development of framework for risk assessment into aiding in the use computational models as diagnostic tools for identifying data gaps and areas of future research to address some of the true model uncertainties.

## Author contributions

AL, KM, NG, JF, and GL are listed as authors. They structured and conducted the analysis; interpreted the simulation results; drafted the manuscript and revised it critically; finally approved the version to be published; and agreed be accountable for all aspects of the work. WT, XY, and FB are acknowledged for their reviewing of this manuscript and their critical comments and edits on the paper.

### Conflict of interest statement

The authors declare that the research was conducted in the absence of any commercial or financial relationships that could be construed as a potential conflict of interest.

## Abbreviations

BBDR: Biologically Based Dose-Response Modeling
PBPK: Physiologically Based Pharmacokinetic
HPT: Hypothalamus-Pituitary-Thyroid Axis
T4: thyroxine
fT4: free thyroxine
T3: tri-iodothyronine
rT3: reverse tri-iodothyronine
eFAST: extended Fourier amplitude sensitivity test
GEM: Gaussian Emulation Machine.
