# Supplementary material for: Quantitative global sensitivity analysis of a biologically based dose-response pregnancy model for the thyroid endocrine system
Source: Front Pharmacol. 2015 May 27;6:107. doi: 10.3389/fphar.2015.00107 (PMC4444753; doi:10.3389/fphar.2015.00107)
Supplement: Supplementary file 1 [file DataSheet1.DOC]

***Supplementary Material***

**Quantitative global sensitivity analysis of a biologically based dose-response pregnancy model for the thyroid endocrine system**

Annie Lumen^1*^, Kevin McNally^3*^, Nysia George^2^, Jeffrey Fisher^1^ and George Loizou^3^

^1^Division of Biochemical Toxicology, National Center for Toxicological Research, U.S. Food and Drug Administration, Jefferson, AR, USA

^2^Division of Bioinformatics and Biostatistics, National Center for Toxicological Research, U.S. Food and Drug Administration, Jefferson, AR, USA

^3^Health and Safety Laboratory, Buxton, Derbyshire, UK.

Correspondence:

*Dr. Annie Lumen

Division of Biochemical Toxicology
U.S. Food and Drug Administration
National Center for Toxicological Research
3900 NCTR Road,

Jefferson, AR, 72079, USA
[annie.lumen@fda.hhs.gov](mailto:annie.lumen@fda.hhs.gov)

&

*Dr. Kevin McNally

C.0.45, Health & Safety Laboratory,

Harpur Hill, Buxton, Derbyshire, SK17 9JN
[kevin.mcnally@hsl.gsi.gov.uk](mailto:kevin.mcnally@hsl.gsi.gov.uk)

1. **Technical appendix**

## Only sparse detail on the emulator-based sensitivity analysis was provided in the main body of the manuscript. This appendix provides additional technical details on the Gaussian Process regression model utilized in this work, and on the interfacing between the acslX and Gaussian Emulation Machine (GEM) software packages. The document describes the underlying concepts rather than the fine technical details. The reader is referred to Oakley and O’Hagan (2004) for underlying mathematics. The document also provides additional details on what the sensitivity measures described in the paper represent and the link to geometry since this allows a better appreciation of how the emulator achieves such massive savings in efficiency.

## Global Sensitivity Analysis

Global Sensitivity Analysis (GSA) aims to quantify the changes in the model output that result from changes to the model inputs. GSA is global in the sense that the inputs are varied over limits defined by probability distributions, as opposed to varied one-at-a-time over an arbitrary and typically small range.

For one or two input dimensions, the changes to the model output in response to perturbations in the inputs can be easily visualized using 2D and 3D plots, respectively. However, for a higher-dimensional vector of inputs, it is difficult to visualize how the output responds to changes in the inputs; therefore, quantitative GSA techniques attempt to explain this variability using various simplified summaries. The approach that is usually adopted for the statistical analysis of highly-multivariate problems is to seek simplifications. For GSA, a commonly adopted simplification is to study the average effect of an input parameter on the model output, or the average effect on the model output as two input parameters are varied at the same time. These are referred to as a *main effect* and a *first-order joint effect* *(or interaction),* which can be visualized using 2D and 3D plots, respectively. This level of simplification is usually justified because high-order interactions between input parameters are often weak compared with main effects and first-order interactions. Many functions can be well approximated as a sum of main effects and first-order interactions. The averages described above are with respect to the redundant input dimensions, which are all but one input and all but two inputs for main effects and first order interactions, respectively. The averages described above are calculated by partial integrals of the output with respect to the model inputs.[[1]](#footnote-2)

A second approach for the GSA of model outputs is variance-based. Variance is a single number that summarizes the total variability in output that results from variation in the inputs. The variance is proportional to the square of the difference between the model output and the mean of all of the model outputs. This can be thought of visually as the square of the area trapped between the curve of the model outputs and the line or plane that represents the mean of all of the model outputs (over the complete input parameter space). The total variance can be decomposed into main-effect variance and interaction variance terms and these also have geometrical interpretations. The main-effect variance is proportional to the squared area trapped between the main effect and the mean of the model output. Figure 1 shows the geometrical link between main effects and their associated variances. The main effect is determined by averaging (by integrating) over all other varying parameters and shows the average change in model output resulting from changes to that one model input. The main effect variance is a single number value that summarizes the main effect and is proportional to the squared areas of the shaded regions in Figure 1. This can be calculated with an additional integral. The main effect cannot be retrieved from the main effect variance. The main effect and main effect variance convey complementary information: the main effect explains how the model output varies (on average) by varying that one parameter; the main-effect variance is the amount of variance that would be removed from the total output variance if the true value of that one parameter was known. There is a similar link between joint effects and the joint effect variance.


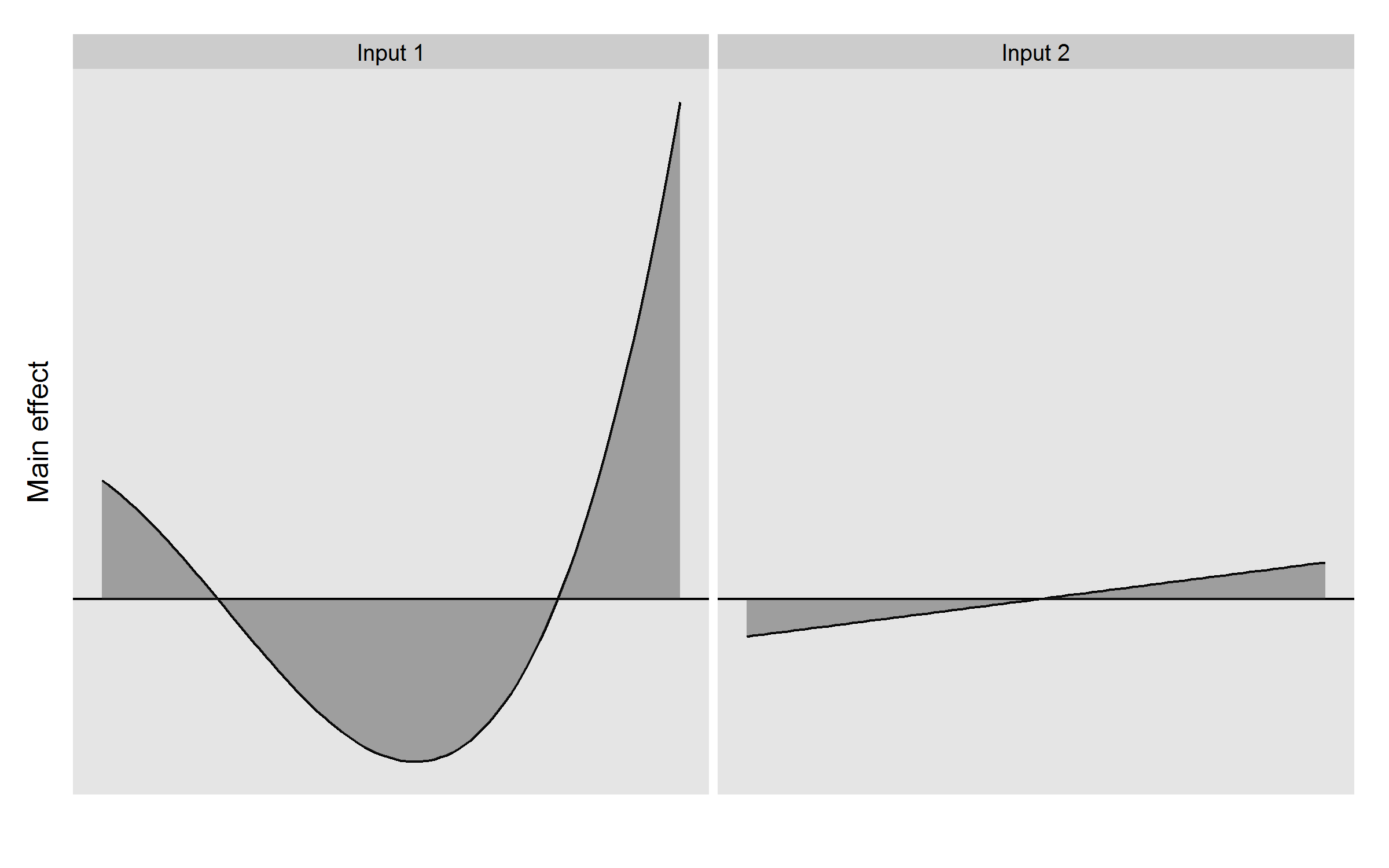


Figure 1: A comparison of two main effect plots. The horizontal line corresponds to the model output mean, and the squared areas, denoted by the shaded regions, are proportional to main effect variances.

For models that consist of simple analytic functions (and common choices of probability distributions), the measures described above can be calculated by analytic integration. For model outputs that require a computational solution, such as the PBPK or BBDR model used in this work (which require a set of ODEs to be numerically solved), analytic integration is not possible. These measures can instead be calculated using numerical integration in this case (for example using Monte Carlo (MC) sampling); however, the sample sizes for reliable results increase rapidly with the number of varying inputs. Depending on the execution time for the computer model, and the number of varying parameters, this may not be computationally feasible.

In practice, main effects and interactions are rarely calculated due to the computational expense of MC sampling. However, more efficient methods, such as eFAST, have been developed for calculating the output variance and main-effect variances (i.e. these can be estimated without the main effects themselves) and the sample sizes are an order of magnitude less than those required for MC. Total-effect variances require larger sample sizes but not excessively so. In the paper, we define the total effect variance as ‘the total effect variance represents the expected amount of output variance that would remain unexplained if only that variable were left free to vary over its range, the value of all other variables being known.’ This is a difficult quantity to understand and clearly not the most informative measure for characterizing the interactions between inputs. Its popularity stems more from the efficiency with which it can be calculated, rather than its information value. However, the information on interactions contained in this measure (which can be assessed by examining the difference between main- and total-effect variances) does supplement the information from main-effect variances, and with expert knowledge on the system being modelled, it can help to interpret the behaviour of the model.

- 1. **Emulator-based GSA**

We have gone into some detail above to describe main effects (and interactions) since we present main-effect plots in results section of the paper and are unaware of these being previously reported in this field; therefore, some additional detail is warranted. The link between main effects and main-effects variances (and similarly interactions) is presented because this allows a better appreciation of how the emulator, as implemented in the GEM software works.

The approach to quantitative global sensitivity analysis in the paper has two phases. The first phase involves the creation of a surrogate model or emulator (technical information on this process follows) that approximates the model output. The second phase involves the calculation of the sensitivity analysis metrics (main effects, main effect variances etc.). GEM uses a particular type of Gaussian Process regression model that allows the analytic calculation of partial integrals with respect to model inputs. Therefore main effects and joint effects can be efficiently calculated (along with a measure of the uncertainty in these estimates). Main-effect and interaction variances can also be estimated as can the total-effect variance. The sensitivity analysis is therefore very rapid. The majority of the computational cost is involved in running the initial computer experiment and building the emulator. Details on the integrals can be found in Oakley and O’Hagan (2004).

GEM supports two classes of probability distribution for the model inputs:

- All parameters are normally distributed (with user-specified mean and standard deviations);
- All parameters uniformly distributed (with user-specified min and max).

In principle, analytical results can be derived with greater flexibility in input distributions (for example, any mixture of uniform, triangular, and normal distributions) and correlated inputs can be accommodated through a multivariate normal distribution. The results presented in the paper assumed all inputs were normally distributed. However, results based upon uniform distributions were similar. Therefore, it is reasonable to conclude that the sensitivity analysis results were insensitive to this modelling choice. Clearly, the probability distribution ascribed to a parameter has little importance if the parameter itself has a negligible effect on the model output.

## Building and validation of the emulator

For the underpinning mathematical development of the GEM Gaussian Process (GP) model, the interested reader is referred to Oakley and O’Hagan (2004). Here we focus on a conceptual description of the model: how it works, the implications, and implicit assumptions.

The Gaussian Process model has elements of global and local regression. A global regression model is specified and this is then corrected for local structure. The emulator mean, which is the central estimate of the model output at any point in input space where a prediction is sought, can be written as


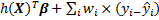
 (1)

The first term in (1) is a parametric approximation to the model output. A general form is written in (1); however, common choices are a constant and a linear term for each input. Both of these forms are accommodated by GEM and, in this work, results were obtained using both as a consistency check. Results from the linear approximation are presented in the paper because this model had a better overall fit although the GSA results were very similar under both model forms.

The estimates of the vector of parameters associated with this regression component of the model in (1) are generalized least squares (GLS) estimates and are calculated using data obtained from a computer experiment: the experiment consisted of *n*= 250 model evaluations in the current work. GLS estimates account for the correlations between the observations; therefore, an *n* by *n* variance-covariance matrix **A** is estimated. In the GP model, the correlations are parameterized. The pairwise correlations between any two sets of outputs for the GP model implemented in GEM take the product form (2), where *p* denotes the number of input dimensions. The correlations between two outputs are expressed in terms of the distance between input points.


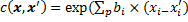
 ) (2)

The parameters *b_i_* are referred to as smoothing parameters and are estimated using data obtained in the computer experiment. These quantify how rapidly the correlation decays to zero with respect to each of the model inputs.

The second term in (1) is where the power of the model lies. This is a weighted sum of the errors from the parametric approximation. These terms ‘correct’ the simple approximation and account for local structure. The weights are a function of input parameters, model outputs and parameters *b_i_* and depend on where in the input space a prediction is sought. The points that are local to where a prediction is sought have greater weighting for a prediction.

Uncertainty in the prediction at any given point is quantified by a standard deviation (formally a *t*-distribution models the uncertainty). This standard deviation is location-dependent reflecting that there is relatively modest uncertainty in the model output very close to points where output has already been observed, and greater uncertainty further away from the design points. A degenerate distribution represents the uncertainty at each design point since the model output is known here with certainty.

The key features of the emulator fit are illustrated in Figure 2 for a simple example with a single input. The parametric approximation was a constant with a single associated parameter: the two panels of Figure 2 show the same input and output data plotted but the central estimate and uncertainty bound (2 standard deviations either side of the mean) shown in the two panels result from two different values for the single smoothing parameter (values of 0.5 and 2, respectively). In application, the smoothing parameter is optimized; however, the two values shown in this example demonstrate the behavior of the correlation function for differing values of the smoothing parameter.


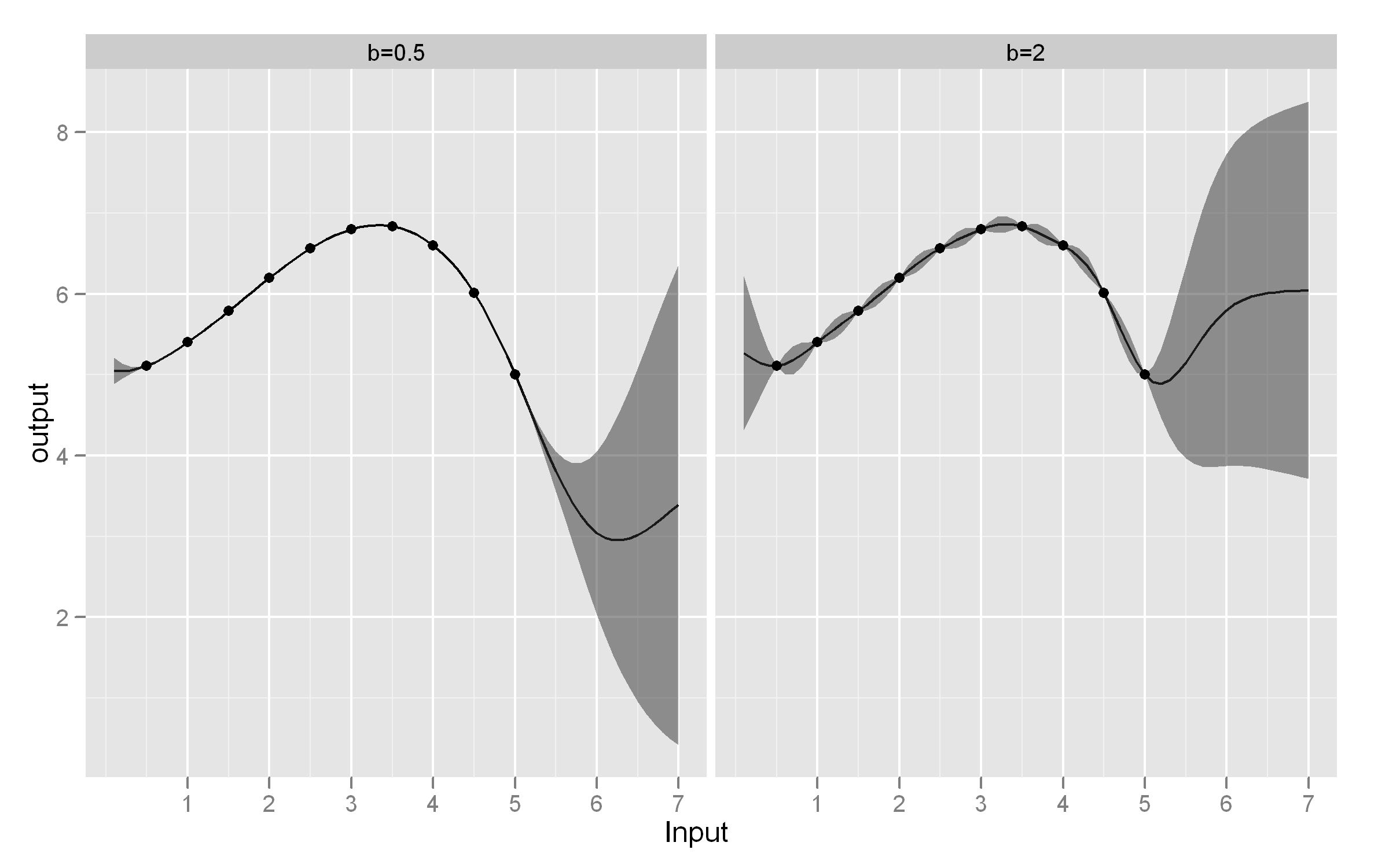


Figure 2: An example of an emulator fit using two varying smoothing parameter for a single input.

In both cases, the central estimate interpolates the data and pinching at the design points can be seen. For the *b* = 2 results, the emulator fit is slightly rougher and the interpolation errors larger compared with *b* = 0.5. However the obvious difference is in the interval surrounding the central estimate. As the correlations become weaker, the prediction uncertainty increases. In both cases, the uncertainty increases beyond the last design points and there is a notable difference in the central estimates beyond an input parameter of 5. Beyond the data from the computer experiment, the correlations weaken and the estimate in Equation 1 is dominated by the first term. This can be seen for *b* = 2, where the central estimate has converged to the regression fit (with associated parameter of approximately 6). Note that had the parametric approximation been a linear fit, then the predictions beyond the data show this linear trend. It is very clear that uncertainties associated with extrapolation are much larger than those associated with interpolation (this is why cross-validation errors, described below, tend to be larger at the ‘edges’ of input space).

In the paper, a BBDR model with 26 uncertain inputs was explored, with a 250 point min-max Latin Hypercube Design (LHD). If all 26 parameters had a strong influence of the model output, such a trivially small number of model evaluations would be insufficient. The power of the Gaussian process emulator lies in the correlations between the inputs and these would be essentially zero. An implicit assumption is, therefore, that some of the parameters have little influence on the model output within the perturbed ranges of the inputs. This appears a strong assumption, but practical experience shows that variability is typically dominated by a small subset of the model parameters. For each dimension of the input vector **x,** the corresponding smoothing parameter *b_i_* is approximately zero if the input has a negligible influence on the output *or* if the parametric approximation is an excellent approximation to the output variation resulting from input *i*. The input space effectively collapses onto a *q < p* dimensional hyperplane. The correlation (2) only depends upon the active dimensions. The min-max LHD is efficient since the coverage of the *q* dimensional hyperplane is adequate and does not require prior knowledge of the redundant dimensions. The result of this reduction in dimensions is that the 250 point design achieves much larger correlations between model outputs than would be expected, given the dimensionality of the BBDR model.

In terms of a reliable sensitivity analysis, the emulator needs to be a sufficiently good approximation of the model such that GSA performed on the emulator can be viewed as an approximation of the analysis on the model. A validation protocol is, therefore, required. It is clear from Figure (2) that the GP model interpolates the data from the computer experiment. As a direct consequence the standard method of model validation, an analysis of residual errors cannot be utilized. Residuals are not defined for an interpolating model. The standard technique for validating an emulator is cross-validation; this is the process of initially fitting the GP model and estimating the associated parameters and then sequentially leaving one point out, and predicting the output using the remaining *n –* 1 design points in order to produce *n* cross validation errors. These errors are subsequently analyzed using similar validation techniques to a multiple linear regression model to assess for overall adequacy and drift.

## Interfacing acslX and GEM

The GSA reported in the paper used the GEM software for the experimental design, emulator fitting, and the calculation of the GSA measures, whereas the model evaluations themselves were done using acslX. This process is referred to as ‘decoupling’. The parameter ranges were specified in GEM, and the LHD was designed and written to a tab delimited text file (250 lines, one parameter set per line). This text file served as an input file to the BBDR model scripted in acslX. Additional scripts were written in acslX to read each line of the input text file that contained a parameter set of 26 values and assign the values to the respective input parameters for each model evaluation. All 250 model evaluations were then run as batch processes in parallel using the high performance scientific computing laboratory clusters at the FDA/Center for Devices and Radiological Health (CDRH)/Office of Science and Engineering Laboratories (OSEL). The model outputs from each of the evaluations were saved as text files. These files were numerically tagged to trace back to the corresponding line of input parameter set from the LHD design input file. The acslX output files were pooled from the cluster for further analysis to ensure correspondence between input set of parameters and the model output. The pooled outputs were also written as a text file, one output per line. Input and output files were read into GEM in order to produce the GP model.

1. **Supplementary Tables**

**Supplementary Table 1.** Results of Morris Screening analysis and sensitivity indices of all model input parameters for each stochastic iteration

|  |  | *Iteration 1* | |  | *Iteration 2* | |  | *Iteration 3* | |
| --- | --- | --- | --- | --- | --- | --- | --- | --- | --- |
| Ranking |  | µ | σ |  | µ | σ |  | µ | σ |
| 1 | KPRODT4F_MI | 0.016848 | 0.000178 | KDEGT4F_MI | 0.016318 | 0.000216 | KDEGT4F_MI | 0.019424 | 0.000313 |
| 2 | KDEGT4F_MI | 0.014936 | 0.000144 | KPRODT4F_MI | 0.01574 | 0.000193 | FRCONVT4_MI | 0.017391 | 0.000235 |
| 3 | FRCONVT4_MI | 0.013761 | 9.21E-05 | FRCONVT4_MI | 0.014736 | 0.000136 | KPRODT4F_MI | 0.016704 | 0.000219 |
| 4 | BW_M | 0.010269 | 5.74E-05 | VDFT4_MI | 0.010568 | 9.13E-05 | BW_M | 0.012258 | 0.000106 |
| 5 | VDFT4_MI | 0.009077 | 5.54E-05 | BW_M | 0.009281 | 6.70E-05 | CLF_UIM | 0.009665 | 0.000109 |
| 6 | CLF_UIM | 0.008365 | 4.88E-05 | CLF_UIM | 0.007858 | 9.72E-05 | VDFT4_MI | 0.009286 | 8.32E-05 |
| 7 | KMNIS_I | 0.007858 | 6.92E-05 | VMAXNISF_THY_MI | 0.00631 | 5.46E-05 | VMAXNISF_THY_MI | 0.00746 | 6.43E-05 |
| 8 | VMAXNISF_THY_MI | 0.006761 | 5.47E-05 | KMNIS_I | 0.005788 | 2.52E-05 | KMNIS_I | 0.007067 | 3.57E-05 |
| 9 | CLFT4_MI | 0.004228 | 4.32E-05 | CLFT4_MI | 0.004988 | 0.000134 | CLFT4_MI | 0.004871 | 3.56E-05 |
| 10 | FRCONVT4_FI | 0.003442 | 1.72E-05 | QFTHY_MI | 0.00306 | 1.52E-05 | FRCONVT4_FI | 0.003607 | 4.34E-05 |
| 11 | QFTHY_MI | 0.002907 | 1.79E-05 | FRCONVT4_FI | 0.00278 | 1.68E-05 | QFTHY_MI | 0.003026 | 1.54E-05 |
| 12 | VDFT4_FI | 0.002698 | 3.02E-05 | PFT4PLC_MI | 0.002165 | 1.92E-05 | VDFT4_FI | 0.002561 | 1.79E-05 |
| 13 | PFT4PLC_MI | 0.002191 | 1.33E-05 | QFC_MI | 0.001812 | 8.07E-06 | PPLCPF_MI | 0.002446 | 1.21E-05 |
| 14 | VMAXNISF_THY_FI | 0.001878 | 9.50E-06 | PAFPLCTTOB_MI | 0.001763 | 9.39E-06 | PFT4PLC_MI | 0.002353 | 1.03E-05 |
| 15 | PAFPLCTTOB_MI | 0.001828 | 5.86E-06 | VDFT4_FI | 0.001612 | 3.32E-06 | PAFPLCTTOB_MI | 0.002191 | 1.49E-05 |
| 16 | PPLCPF_MI | 0.001777 | 7.04E-06 | VMAXNISF_THY_FI | 0.001458 | 5.38E-06 | VMAXNISF_THY_FI | 0.001958 | 1.03E-05 |
| 17 | PPLC_MI | 0.001681 | 1.00E-05 | PPLCPF_MI | 0.001447 | 6.94E-06 | QFC_MI | 0.00186 | 8.45E-06 |
| 18 | PAFT4PLCF_MI | 0.001639 | 1.67E-05 | KDEGT4F_FI | 0.001158 | 2.70E-06 | PAFT4PLCF_MI | 0.001573 | 5.26E-06 |
| 19 | QFC_MI | 0.001502 | 3.03E-06 | PPLC_MI | 0.001125 | 2.73E-06 | PPLC_MI | 0.001385 | 3.37E-06 |
| 20 | KDEGT4F_FI | 0.001254 | 6.34E-06 | PAFPLCBTOT_MI | 0.001098 | 2.86E-06 | PAFPLCBTOT_MI | 0.00118 | 2.17E-06 |
| 21 | PAFPLCBTOT_MI | 0.001182 | 3.88E-06 | PAFT4PLCF_MI | 0.001029 | 1.42E-06 | KDEGT4F_FI | 0.001152 | 1.74E-06 |
| 22 | KPRODT4F_FI | 0.000952 | 2.08E-05 | KPRODT4F_FI | 0.000779 | 2.65E-06 | QFRP_MI | 0.000783 | 1.22E-06 |
| 23 | QFC_FI | 0.000833 | 1.46E-05 | QFRP_MI | 0.000742 | 6.60E-07 | VMAXNISF_PLC_MI | 0.000709 | 2.33E-06 |
| 24 | QFRP_MI | 0.000687 | 1.37E-06 | VMAXNISF_PLC_MI | 0.000556 | 1.90E-06 | KPRODT4F_FI | 0.000665 | 1.94E-06 |
| 25 | QFTHY_FI | 0.00068 | 1.49E-06 | KPRODT3F_MI | 0.000505 | 1.38E-07 | QFC_FI | 0.00063 | 1.65E-06 |
| 26 | VMAXNISF_PLC_MI | 0.000646 | 1.90E-06 | QFTHY_FI | 0.0005 | 7.82E-07 | QFTHY_FI | 0.000621 | 1.61E-06 |
| 27 | KPRODT3F_MI | 0.000537 | 1.87E-07 | QFC_FI | 0.000477 | 1.76E-06 | KPRODT3F_MI | 0.00053 | 1.26E-07 |
| 28 | BW_F | 0.000503 | 1.00E-06 | BW_F | 0.000465 | 4.76E-07 | BW_F | 0.000449 | 3.88E-07 |
| 29 | QFROB_FI | 0.000316 | 6.52E-07 | QFROB_FI | 0.000324 | 3.18E-07 | QFROB_FI | 0.000399 | 6.32E-07 |
| 30 | QFSP_MI | 0.000284 | 9.47E-08 | QFSP_MI | 0.000302 | 1.35E-07 | QFSP_MI | 0.000317 | 1.39E-07 |
| 31 | QFPLC_MI | 0.000222 | 1.10E-07 | QFPLC_MI | 0.000255 | 1.90E-07 | QFPLC_MI | 0.000254 | 1.03E-07 |
| 32 | IODSTORES_MG_FI | 0.000118 | 1.44E-08 | KDEGT3F_MI | 0.000158 | 2.05E-07 | IODSTORES_MG_FI | 0.000151 | 2.82E-08 |
| 33 | KDEGT3F_MI | 0.000106 | 3.09E-08 | IODSTORES_MG_FI | 0.000139 | 2.18E-08 | CLFT3_MI | 0.000137 | 6.47E-08 |
| 34 | CLFT3_MI | 0.000104 | 1.55E-08 | VDFT3_MI | 0.000121 | 1.32E-07 | KDEGT3F_MI | 0.000112 | 3.93E-08 |
| 35 | KPRODT3F_FI | 7.98E-05 | 1.11E-08 | CLFT3_MI | 0.000106 | 4.44E-08 | KPRODT3F_FI | 9.15E-05 | 1.71E-08 |
| 36 | VFRP_MI | 5.61E-05 | 4.94E-09 | KPRODT3F_FI | 7.85E-05 | 1.07E-08 | VDFT3_MI | 8.43E-05 | 3.04E-08 |
| 37 | VDFT3_MI | 5.03E-05 | 4.75E-09 | VFRP_MI | 7.44E-05 | 9.49E-09 | VFRP_MI | 6.53E-05 | 3.18E-09 |
| 38 | VFPLC_MI | 3.49E-05 | 8.41E-10 | VFPLC_MI | 4.10E-05 | 2.16E-09 | VFPLC_MI | 3.74E-05 | 1.40E-09 |
| 39 | VFPLCT_MI | 2.65E-05 | 3.92E-09 | VFPLCT_MI | 2.58E-05 | 6.06E-09 | VFSP_MI | 1.60E-05 | 9.13E-10 |
| 40 | VFSP_MI | 1.59E-05 | 5.74E-10 | VFSP_MI | 1.58E-05 | 5.12E-10 | VFPLCT_MI | 1.46E-05 | 8.29E-10 |
| 41 | PRP_MI | 6.90E-06 | 9.35E-11 | VFPLCB_MI | 7.09E-06 | 1.47E-09 | PRP_MI | 7.87E-06 | 1.68E-10 |
| 42 | PAFTHY_MI | 6.61E-06 | 1.15E-10 | PRP_MI | 5.85E-06 | 1.04E-10 | PAFTHY_MI | 6.03E-06 | 1.17E-10 |
| 43 | VFPLCB_MI | 3.95E-06 | 5.70E-11 | PAFTHY_MI | 3.83E-06 | 5.32E-11 | VFPLCB_MI | 5.68E-06 | 1.04E-10 |
| 44 | PSP_MI | 3.32E-06 | 2.89E-11 | PSP_MI | 2.85E-06 | 1.73E-11 | PROB_FI | 4.19E-06 | 8.76E-11 |
| 45 | PROB_FI | 2.11E-06 | 2.32E-11 | VFPLS_MI | 2.39E-06 | 1.22E-11 | PSP_MI | 3.35E-06 | 1.96E-11 |
| 46 | VFPLS_MI | 1.93E-06 | 6.74E-12 | PROB_FI | 2.13E-06 | 1.42E-11 | VFPLS_MI | 2.27E-06 | 7.38E-12 |
| 47 | TLEN_I | 9.74E-07 | 1.30E-12 | TLEN_I | 8.67E-07 | 2.28E-12 | PAFTHY_FI | 1.53E-06 | 2.08E-11 |
| 48 | PAFTHY_FI | 9.13E-07 | 2.84E-12 | PAFTHY_FI | 8.12E-07 | 1.78E-12 | TLEN_I | 1.16E-06 | 2.25E-12 |
| 49 | KDEGT3F_FI | 4.69E-07 | 2.76E-12 | KDEGT3F_FI | 6.93E-07 | 3.08E-12 | KDEGT3F_FI | 3.44E-07 | 5.53E-13 |
| 50 | VFPLS_FI | 1.10E-07 | 3.86E-14 | VFPLS_FI | 1.14E-07 | 6.69E-14 | VFPLS_FI | 1.05E-07 | 8.66E-14 |
| 51 | VFROB_FI | 3.63E-08 | 3.91E-15 | VFROB_FI | 3.72E-08 | 4.24E-15 | VFROB_FI | 3.48E-08 | 4.94E-15 |
| 52 | VFTHY_MI | 1.31E-08 | 2.60E-16 | VFTHY_MI | 2.00E-08 | 9.89E-16 | VFTHY_MI | 2.01E-08 | 8.75E-16 |
| 53 | VFTHYB_MI | 6.91E-09 | 1.45E-16 | VFTHYB_MI | 7.30E-09 | 2.20E-16 | VFTHYB_MI | 5.67E-09 | 9.01E-17 |
| 54 | VFTHY_FI | 2.85E-09 | 2.85E-17 | CLF_BIND_MI | 2.45E-09 | 1.65E-17 | VFTHY_FI | 4.90E-09 | 1.90E-16 |
| 55 | CLF_BIND_MI | 1.99E-09 | 1.36E-17 | VFTHY_FI | 2.26E-09 | 2.26E-17 | PTHY_MI | 2.20E-09 | 2.88E-17 |
| 56 | VFTHYB_FI | 1.64E-09 | 7.04E-18 | VFTHYB_FI | 1.99E-09 | 2.09E-17 | CLF_BIND_MI | 1.78E-09 | 9.27E-18 |
| 57 | PTHY_MI | 1.27E-09 | 3.00E-18 | PTHY_MI | 1.54E-09 | 7.20E-18 | VFTHYB_FI | 1.41E-09 | 9.45E-18 |
| 58 | CLF_BIND_FI | 8.51E-10 | 2.66E-18 | CLF_BIND_FI | 4.51E-10 | 6.18E-19 | CLF_BIND_FI | 1.01E-09 | 2.00E-17 |
| 59 | PTHY_FI | 6.73E-10 | 5.63E-18 | PTHY_FI | 3.71E-10 | 9.55E-19 | PTHY_FI | 7.20E-10 | 2.67E-18 |
| 60 | VFTHYT_FI | 9.63E-11 | 1.13E-19 | VFTHYT_FI | 2.53E-10 | 4.57E-18 | VFTHYT_FI | 1.12E-10 | 2.28E-19 |
| 61 | VFTHYT_MI | 3.75E-11 | 2.99E-20 | VFTHYT_MI | 6.58E-11 | 7.96E-20 | VFTHYT_MI | 5.99E-11 | 9.23E-20 |
| 62 | IODSTORES_MG_MI | 0 | 0 | IODSTORES_MG_MI | 0 | 0 | IODSTORES_MG_MI | 0 | 0 |
| 63 | VDFT3_FI | 0 | 0 | VDFT3_FI | 0 | 0 | VDFT3_FI | 0 | 0 |
| 64 | VURINE | 0 | 0 | VURINE | 0 | 0 | VURINE | 0 | 0 |
| 65 | PAFPLC_MI | 0 | 0 | PAFPLC_MI | 0 | 0 | PAFPLC_MI | 0 | 0 |
| 66 | PTT4PLC_MI | 0 | 0 | PTT4PLC_MI | 0 | 0 | PTT4PLC_MI | 0 | 0 |

**Supplementary Table 2.** Comparison of the ranking of model parameters based on the two Morris screening sensitivity indices

|  | a) Parameter Ranking (µ) | | |  | b) Parameter Ranking (σ) | | |
| --- | --- | --- | --- | --- | --- | --- | --- |
|  | *Iteration 1* | *Iteration 2* | *Iteration 3* |  | *Iteration 1* | *Iteration 2* | *Iteration 3* |
| R#1 | KPRODT4F_MI | KDEGT4F_MI | KDEGT4F_MI | R#1 | KPRODT4F_MI | KDEGT4F_MI | KDEGT4F_MI |
| R#2 | KDEGT4F_MI | KPRODT4F_MI | FRCONVT4_MI | R#2 | KDEGT4F_MI | KPRODT4F_MI | FRCONVT4_MI |
| R#3 | FRCONVT4_MI | FRCONVT4_MI | KPRODT4F_MI | R#3 | FRCONVT4_MI | FRCONVT4_MI | KPRODT4F_MI |
| R#4 | BW_M | VDFT4_MI | BW_M | R#4 | KMNIS_I | CLFT4_MI | CLF_UIM |
| R#5 | VDFT4_MI | BW_M | CLF_UIM | R#5 | BW_M | CLF_UIM | BW_M |
| R#6 | CLF_UIM | CLF_UIM | VDFT4_MI | R#6 | VDFT4_MI | VDFT4_MI | VDFT4_MI |
| R#7 | KMNIS_I | VMAXNISF_THY_MI | VMAXNISF_THY_MI | R#7 | VMAXNISF_THY_MI | BW_M | VMAXNISF_THY_MI |
| R#8 | VMAXNISF_THY_MI | KMNIS_I | KMNIS_I | R#8 | CLF_UIM | VMAXNISF_THY_MI | FRCONVT4_FI |
| R#9 | CLFT4_MI | CLFT4_MI | CLFT4_MI | R#9 | CLFT4_MI | KMNIS_I | KMNIS_I |
| R#10 | FRCONVT4_FI | QFTHY_MI | FRCONVT4_FI | R#10 | VDFT4_FI | PFT4PLC_MI | CLFT4_MI |
| R#11 | QFTHY_MI | FRCONVT4_FI | QFTHY_MI | R#11 | KPRODT4F_FI | FRCONVT4_FI | VDFT4_FI |
| R#12 | VDFT4_FI | PFT4PLC_MI | VDFT4_FI | R#12 | QFTHY_MI | QFTHY_MI | QFTHY_MI |
| R#13 | PFT4PLC_MI | QFC_MI | PPLCPF_MI | R#13 | FRCONVT4_FI | PAFPLCTTOB_MI | PAFPLCTTOB_MI |
| R#14 | VMAXNISF_THY_FI | PAFPLCTTOB_MI | PFT4PLC_MI | R#14 | PAFT4PLCF_MI | QFC_MI | PPLCPF_MI |
| R#15 | PAFPLCTTOB_MI | VDFT4_FI | PAFPLCTTOB_MI | R#15 | QFC_FI | PPLCPF_MI | VMAXNISF_THY_FI |
| R#16 | PPLCPF_MI | VMAXNISF_THY_FI | VMAXNISF_THY_FI | R#16 | PFT4PLC_MI | VMAXNISF_THY_FI | PFT4PLC_MI |
| R#17 | PPLC_MI | PPLCPF_MI | QFC_MI | R#17 | PPLC_MI | VDFT4_FI | QFC_MI |
| R#18 | PAFT4PLCF_MI | KDEGT4F_FI | PAFT4PLCF_MI | R#18 | VMAXNISF_THY_FI | PAFPLCBTOT_MI | PAFT4PLCF_MI |
| R#19 | QFC_MI | PPLC_MI | PPLC_MI | R#19 | PPLCPF_MI | PPLC_MI | PPLC_MI |
| R#20 | KDEGT4F_FI | PAFPLCBTOT_MI | PAFPLCBTOT_MI | R#20 | KDEGT4F_FI | KDEGT4F_FI | VMAXNISF_PLC_MI |
| R#21 | PAFPLCBTOT_MI | PAFT4PLCF_MI | KDEGT4F_FI | R#21 | PAFPLCTTOB_MI | KPRODT4F_FI | PAFPLCBTOT_MI |
| R#22 | KPRODT4F_FI | KPRODT4F_FI | QFRP_MI | R#22 | PAFPLCBTOT_MI | VMAXNISF_PLC_MI | KPRODT4F_FI |
| R#23 | QFC_FI | QFRP_MI | VMAXNISF_PLC_MI | R#23 | QFC_MI | QFC_FI | KDEGT4F_FI |
| R#24 | QFRP_MI | VMAXNISF_PLC_MI | KPRODT4F_FI | R#24 | VMAXNISF_PLC_MI | PAFT4PLCF_MI | QFC_FI |
| R#25 | QFTHY_FI | KPRODT3F_MI | QFC_FI | R#25 | QFTHY_FI | QFTHY_FI | QFTHY_FI |
| R#26 | VMAXNISF_PLC_MI | QFTHY_FI | QFTHY_FI | R#26 | QFRP_MI | QFRP_MI | QFRP_MI |
| R#27 | KPRODT3F_MI | QFC_FI | KPRODT3F_MI | R#27 | BW_F | BW_F | QFROB_FI |
| R#28 | BW_F | BW_F | BW_F | R#28 | QFROB_FI | QFROB_FI | BW_F |
| R#29 | QFROB_FI | QFROB_FI | QFROB_FI | R#29 | KPRODT3F_MI | KDEGT3F_MI | QFSP_MI |
| R#30 | QFSP_MI | QFSP_MI | QFSP_MI | R#30 | QFPLC_MI | QFPLC_MI | KPRODT3F_MI |
| R#31 | QFPLC_MI | QFPLC_MI | QFPLC_MI | R#31 | QFSP_MI | KPRODT3F_MI | QFPLC_MI |
| R#32 | IODSTORES_MG_FI | KDEGT3F_MI | IODSTORES_MG_FI | R#32 | KDEGT3F_MI | QFSP_MI | CLFT3_MI |
| R#33 | KDEGT3F_MI | IODSTORES_MG_FI | CLFT3_MI | R#33 | CLFT3_MI | VDFT3_MI | KDEGT3F_MI |
| R#34 | CLFT3_MI | VDFT3_MI | KDEGT3F_MI | R#34 | IODSTORES_MG_FI | CLFT3_MI | VDFT3_MI |
| R#35 | KPRODT3F_FI | CLFT3_MI | KPRODT3F_FI | R#35 | KPRODT3F_FI | IODSTORES_MG_FI | IODSTORES_MG_FI |
| R#36 | VFRP_MI | KPRODT3F_FI | VDFT3_MI | R#36 | VFRP_MI | KPRODT3F_FI | KPRODT3F_FI |
| R#37 | VDFT3_MI | VFRP_MI | VFRP_MI | R#37 | VDFT3_MI | VFRP_MI | VFRP_MI |
| R#38 | VFPLC_MI | VFPLC_MI | VFPLC_MI | R#38 | VFPLCT_MI | VFPLCT_MI | VFPLC_MI |
| R#39 | VFPLCT_MI | VFPLCT_MI | VFSP_MI | R#39 | VFPLC_MI | VFPLC_MI | VFSP_MI |
| R#40 | VFSP_MI | VFSP_MI | VFPLCT_MI | R#40 | VFSP_MI | VFPLCB_MI | VFPLCT_MI |
| R#41 | PRP_MI | VFPLCB_MI | PRP_MI | R#41 | PAFTHY_MI | VFSP_MI | PRP_MI |
| R#42 | PAFTHY_MI | PRP_MI | PAFTHY_MI | R#42 | PRP_MI | PRP_MI | PAFTHY_MI |
| R#43 | VFPLCB_MI | PAFTHY_MI | VFPLCB_MI | R#43 | VFPLCB_MI | PAFTHY_MI | VFPLCB_MI |
| R#44 | PSP_MI | PSP_MI | PROB_FI | R#44 | PSP_MI | PSP_MI | PROB_FI |
| R#45 | PROB_FI | VFPLS_MI | PSP_MI | R#45 | PROB_FI | PROB_FI | PAFTHY_FI |
| R#46 | VFPLS_MI | PROB_FI | VFPLS_MI | R#46 | VFPLS_MI | VFPLS_MI | PSP_MI |
| R#47 | TLEN_I | TLEN_I | PAFTHY_FI | R#47 | PAFTHY_FI | KDEGT3F_FI | VFPLS_MI |
| R#48 | PAFTHY_FI | PAFTHY_FI | TLEN_I | R#48 | KDEGT3F_FI | TLEN_I | TLEN_I |
| R#49 | KDEGT3F_FI | KDEGT3F_FI | KDEGT3F_FI | R#49 | TLEN_I | PAFTHY_FI | KDEGT3F_FI |
| R#50 | VFPLS_FI | VFPLS_FI | VFPLS_FI | R#50 | VFPLS_FI | VFPLS_FI | VFPLS_FI |
| R#51 | VFROB_FI | VFROB_FI | VFROB_FI | R#51 | VFROB_FI | VFROB_FI | VFROB_FI |
| R#52 | VFTHY_MI | VFTHY_MI | VFTHY_MI | R#52 | VFTHY_MI | VFTHY_MI | VFTHY_MI |
| R#53 | VFTHYB_MI | VFTHYB_MI | VFTHYB_MI | R#53 | VFTHYB_MI | VFTHYB_MI | VFTHY_FI |
| R#54 | VFTHY_FI | CLF_BIND_MI | VFTHY_FI | R#54 | VFTHY_FI | VFTHY_FI | VFTHYB_MI |
| R#55 | CLF_BIND_MI | VFTHY_FI | PTHY_MI | R#55 | CLF_BIND_MI | VFTHYB_FI | PTHY_MI |
| R#56 | VFTHYB_FI | VFTHYB_FI | CLF_BIND_MI | R#56 | VFTHYB_FI | CLF_BIND_MI | CLF_BIND_FI |
| R#57 | PTHY_MI | PTHY_MI | VFTHYB_FI | R#57 | PTHY_FI | PTHY_MI | VFTHYB_FI |
| R#58 | CLF_BIND_FI | CLF_BIND_FI | CLF_BIND_FI | R#58 | PTHY_MI | VFTHYT_FI | CLF_BIND_MI |
| R#59 | PTHY_FI | PTHY_FI | PTHY_FI | R#59 | CLF_BIND_FI | PTHY_FI | PTHY_FI |
| R#60 | VFTHYT_FI | VFTHYT_FI | VFTHYT_FI | R#60 | VFTHYT_FI | CLF_BIND_FI | VFTHYT_FI |
| R#61 | VFTHYT_MI | VFTHYT_MI | VFTHYT_MI | R#61 | VFTHYT_MI | VFTHYT_MI | VFTHYT_MI |
| R#62 | IODSTORES_MG_MI | IODSTORES_MG_MI | IODSTORES_MG_MI | R#62 | IODSTORES_MG_MI | IODSTORES_MG_MI | IODSTORES_MG_MI |
| R#63 | VDFT3_FI | VDFT3_FI | VDFT3_FI | R#63 | VDFT3_FI | VDFT3_FI | VDFT3_FI |
| R#64 | VURINE | VURINE | VURINE | R#64 | VURINE | VURINE | VURINE |
| R#65 | PAFPLC_MI | PAFPLC_MI | PAFPLC_MI | R#65 | PAFPLC_MI | PAFPLC_MI | PAFPLC_MI |
| R#66 | PTT4PLC_MI | PTT4PLC_MI | PTT4PLC_MI | R#66 | PTT4PLC_MI | PTT4PLC_MI | PTT4PLC_MI |

1. This is a key difference compared with local sensitivity analysis, which fixes all the other inputs at baseline values whilst varying a single parameter over a small range. [↑](#footnote-ref-2)
